# Transient intracranial pressure elevations (B waves) are associated with sleep apnea

**DOI:** 10.1186/s12987-023-00469-6

**Published:** 2023-10-02

**Authors:** Casper Schwartz Riedel, Isabel Martinez-Tejada, Morten Andresen, Jens E. Wilhjelm, Poul Jennum, Marianne Juhler

**Affiliations:** 1 Department of Neurosurgery, Copenhagen University Hospital, Rigshospitalet, Inge Lehmanns Vej 6, Copenhagen East, 2100 Copenhagen, Denmark; 2 Danish Center for Sleep Medicine, Department of Clinical Neurophysiology, Copenhagen University Hospital, Rigshospitalet, Glostrup, Denmark; 3 Department of Health Technology, Technical University of Denmark, Kongens Lyngby, Denmark; 4 Institute of Clinical Medicine, University of Copenhagen, Copenhagen, Denmark

**Keywords:** Obstructive sleep apnea, Sleep-disordered breathing, CPAP, ICP, Slow waves, Hydrocephalus

## Abstract

**Background:**

Repetitive transient intracranial pressure waveform elevations up to 50 mmHg (ICP B-waves) are often used to define pathological conditions and determine indications for ICP-reducing treatment. We recently showed that nocturnal transient ICP elevations are present in patients without structural brain lesions or hydrocephalus in whom they are associated with sleep apnea. However, whether this signifies a general association between ICP macropatterns and sleep apnea remains unknown.

**Methods:**

We included 34 patients with hydrocephalus, or idiopathic intracranial hypertension (IIH), who were referred to the Neurosurgical Department, Copenhagen, Denmark, from 2017 to 2021. Every patient underwent diagnostic overnight ICP monitoring for clinical indications, with simultaneous polysomnography (PSG) sleep studies. All transient ICP elevations were objectively quantified in all patients. Three patients were monitored with continuous positive airway pressure (CPAP) treatment for an additional night.

**Results:**

All patients had transient ICP elevations associated with sleep apnea. The mean temporal delay from sleep apnea to transient ICP elevations for all patients was 3.6 s (SEM 0.2 s). Ramp-type transient ICP elevations with a large increase in ICP were associated with rapid eye movement (REM) sleep and sinusoidal-type elevations with non-REM (NREM) sleep. In three patients treated with CPAP, the treatment reduced the number of transient ICP elevations with a mean of 37%. CPAP treatment resulted in insignificant changes in the average ICP in two patients but elevated the average ICP during sleep in one patient by 5.6 mmHg.

**Conclusion:**

The findings suggest that sleep apnea causes a significant proportion of transient ICP elevations, such as B-waves, and sleep apnea should be considered in ICP evaluation. Treatment of sleep apnea with CPAP can reduce the occurrence of transient ICP elevations. More research is needed on the impact of slow oscillating mechanisms on transient ICP elevations during high ICP and REM sleep.

**Supplementary Information:**

The online version contains supplementary material available at 10.1186/s12987-023-00469-6.

## Background

Intracranial pressure (ICP) measurement is a cornerstone of diagnostics in neurology and neurosurgery. One significant aspect of ICP analysis is the characterization of different macropatterns, such as transient ICP elevations. Lundberg initially described different macropatterns of ICP and categorized them as A-, B-, and C-waves based on frequency, amplitude, and duration [1]. This paper centers on Lundberg B-waves, which exhibit frequencies of 0.5 to 2 waves per minute and amplitudes up to 50 mmHg. These B-waves are the most clinically used form of transient ICP elevations and guide decisions for invasive ICP management.

Historically, transient ICP elevations such as B-waves have been considered signs of reduced intracranial compliance. Despite their use in guiding decisions for invasive ICP management, the underlying physiology of these transient elevations remains elusive. Previous studies have reported their occurrence in healthy individuals [2–4], and they are associated with sleep-disordered breathing, such as sleep apnea [1, 4–7]. However, this has not attracted much attention, and transient ICP elevations are generally considered a result of altered cerebral blood flow (CBF) due to changes in blood gases, especially CO_2_. We recently showed that B-waves are associated with sleep apnea, and that ramp-like transient ICP elevations are associated with a large increase in the ICP with rapid eye movement (REM) sleep. In contrast, more minor sinusoidal increases in ICP primarily occurred with non-REM (NREM) sleep stages [4]. Additionally, we suggested that pressure changes in the thoracic cavity caused by respiratory movement and sleep apnea could contribute to a significant proportion of transient ICP elevations.

Several underlying physiological mechanisms probably cause transient ICP elevations, depending on ICP level and clinical conditions. We propose that there is a strong connection between transient ICP elevations and sleep-disordered breathing, such as sleep apnea, in patients with idiopathic normal pressure hydrocephalus (iNPH). Both B-waves and sleep apnea are highly prevalent in these patients [8–13]. We also propose similar patterns in patients with other neurosurgical conditions.

By exploring the connection between transient ICP elevations, sleep stages, and sleep apnea, this study aims to provide valuable insights into a better understanding of the underlying mechanisms of transient ICP elevations. This was done by recording ICP signals and conducting simultaneous polysomnography sleep studies in 34 patients with different types of hydrocephalus and idiopathic intracranial hypertension (IIH). The study also examined the effects of continuous positive airway pressure (CPAP) treatment on the number of sleep apnea-associated transient ICP elevations by monitoring three patients for an additional night when treated with CPAP.

## Methods

This prospective observational study was conducted and reported according to the STROBE guidelines for cohort studies. It focuses mainly on the nocturnal occurrence of transient ICP elevations in neurosurgical patients and is part of a more extensive research program investigating sleep and the glymphatic system in humans.

### Study population

The cohort included 34 patients with hydrocephalus or IIH referred to the Neurosurgical Department, Copenhagen, Denmark, from 2017 to 2021. To confirm the diagnosis, every patient underwent a physical, neurological, and radiological evaluation. ICP monitoring was performed for diagnostic purposes. The patients were asked to participate in an additional voluntary sleep evaluation study performed together with ICP monitoring. Upon enrollment, a hydrocephalus expert classified the patients with the following diagnoses: iNPH, adult-onset obstructive hydrocephalus, pediatric-onset hydrocephalus, following the ASPECT Hydrocephalus System for evaluation of hydrocephalus patients [14] or IIH (Table 1).

**Table 1:** Demographic and clinical data

	Type of patient				
	iNPH (*n* = 5)	Adult-onset (*n* = 4)	Pediatric-onset (*n* = 13)	IIH (*n* = 12)	*P* value
Men/Women	4 (20%)/1 (20%)	1 (25%)/3 (75%)	5 (45%)/6 (55%)	1 (8%)/11 (92%)	
Age, years	67.4 (6.6)	60.0 (7.2)	42.9 (20.5)	33.0 (8.0)	**< 0.001**
BMI	25.4 (3.4)	26.1 (3.0)	26.5 (6.4)	36.5 (9.6)	**0.005**
ESS	4.8 (3.1)	7.8 (5.3)	10.5 (6.1)	9.1 (4.3)	0.23
General disease and risk factors					
Hypertension	4 (80%)	1 (25%)	1 (8%)	3 (25%)	
Heart disease	3 (60%)	0	0	1 (10%)	
CNS					
Dementia	4 (80%)	0	3 (23%)	0	
Psychiatric	0	0	0	2 (17%)	
Epilepsy	2 (40%)	1 (25%)	3 (23%)	0	
Evans ratio	0.44 (0.05)	0.33 (0.08)	0.34 (0.07)	0.26 (0.04)	**< 0.001**
DESH	3 (60%)	0	1 (8%)	0	
Obstruction	0	4 (100%)	9 (69%)	0	
Treatment					
Shunt	0	4 (100%)	2 (15%)	4 (33%)	
ETV	1 (20%)	1 (25%)	7 (54%)	0	

Summary statistics include the mean and the standard deviation (SD) to summarize continuous variables and numbers and (percentages) to summarize categorical variables

*BMI* body mass index (kg/m^2^), *DESH* disproportionately enlarged subarachnoid space hydrocephalus, Epworth sleepiness scale (ESS, normal < 10)), *ETV* endoscopic third ventriculostomy, *Evans ratio* ventricular size ratio (normal ≤ 0.30), *SD* standard deviation

*P* value of one-way analysis of variance (ANOVA)

The iNPH diagnosis was based on the iNPH symptom triad and imaging. None of the iNPH patients had a previous history of tumors, subarachnoid hemorrhage, head injury, or meningitis and had no visible obstruction of CSF flow on imaging. All adult-onset hydrocephalus patients showed signs of CSF flow obstruction on imaging. The pediatric-onset patients had a documented pediatric-onset or a clinical presentation compatible with pediatric-onset, e.g., congenital malformations and a large head circumference. The patients with IIH were diagnosed based on the Friedman criteria with normal brain parenchyma without hydrocephalus, mass, or structural lesion and no abnormal meningeal enhancement or venous sinus thrombosis [15]. Three patients with IIH were included upon an acute diagnosis with papilledema. The demographic and clinical data of the patient groups are summarized in Table 1. Except for one patient with pediatric-onset hydrocephalus, the prevalence of sleep apnea has been reported previously for all patients with hydrocephalus in this cohort [8].

### Surgery and ICP catheter

All patients underwent surgery by inserting a commercially available ICP catheter (RAUMEDIC AG, Helmbrechts, Germany). Fourteen patients were measured with a Neurovent-P catheter and seven with Neurovent-P-tel catheter (telemetric catheter), both inserted 2 cm into the brain parenchyma in the right frontal region (Kocher's point) and measuring the ICP in the parenchyma. The remaining nine patients were measured with a Neurovent catheter, measuring ICP in the lateral ventricle (Additional file 1: Table S1). All operations were completed without complications, and all patients were discharged after their diagnostic ICP measurement or the next day for patients with Neurovent-P-tel. Calibration is not necessary before insertion, as these systems autocalibrate against atmospheric pressure.

### PSG

As previously described, the sleep evaluation was performed during in-hospital diagnostics with ICP measurements [4]. Polysomnography (PSG) is a standard criterion method and was performed according to the American Academy of Sleep Medicine (AASM) standards [electroencephalogram (EEG), electrooculography (EOG), submental- and anterior tibialis electromyography (EMG), electrocardiography (ECG), airflow, respiratory inductance plethysmography (RIP), snoring, and oxyhemoglobin saturation (SaO_2_)], and was recorded with Domino™ software (SOMNOmedics GmbH, Germany). Recordings were scored manually according to the AASM by an independent trained professional supervised by an experienced expert in neurophysiology, both blinded to the diagnosis of the patients. Sleep-disordered breathing is reported as the apnea–hypopnea index (AHI) following the AASM 2012 standard. In addition, two patients with IIH and two with pediatric-onset were monitored with end-tidal CO_2_ (LoFlo, Respironics, Inc.) according to the manufacturer's protocol.

### CPAP

Three patients with sleep apnea were monitored for two days with ICP and PSG. Continuous positive airway pressure (CPAP) treatment was added for the second night of monitoring. None of the three patients were known to have sleep apnea prior to our investigation and had not tried CPAP treatment before.

### ICP and PSG recordings

As previously described [4], simultaneous recordings of PSG (SOMNOscreen™ plus, Somnomedics) and ICP (RAUMEDIC AG) were performed. Briefly, after manually scoring the PSG, all data were transferred to MATLAB 2020b (MathWorks^®^). We recorded data using two sensors: the Datalogger MPR1 and the PSG. Since these sensors collected data asynchronously, time alignment was necessary to eliminate delays. Furthermore, this also ensured the accuracy of absolute ICP values by avoiding drift of the ICP signal recorded by the PSG. We achieved this alignment by integrating the Datalogger MPR1 signal into the PSG signal using MATLAB. Specifically, we used the transferred ICP signal record by the PSG as a temporal reference, guiding the alignment process to ensure that each peak and pattern in the ICP signal matched. Subsequent ICP analyses exclusively utilized the synchronized ICP data from the Datalogger. Before conducting the ICP analysis, artifacts were identified and removed from the synchronized ICP signal.

### Transient ICP elevations

All transient ICP elevations were identified by the recently described method for automatic macropattern identification in the ICP signal [16] and subdivided into those occurring during REM sleep vs. NREM sleep and into those occurring with or without apnea or hypopnea. Briefly, artifacts were removed with an empirical decomposition (EMD) based method. Afterward, to avoid the algorithm from detecting normal variation in the ICP signal from respiration and cardiac pulsation, the ICP signal was smoothed via a linear phase FIR lowpass filter, with a cut-off frequency (Fpass) set to 0.05–0.1 Hz (for details and examples of raw and smoothed ICP, see Martinez‑Tejada et al*.* [16]). Then the ICP signal was segmented into subsequences of duration varying from seconds to minutes by finding the local maxima and minima in the smoothed ICP signal. The derived subsequences were Z-normalized to ensure that the subsequences were linearly transformed to have a zero mean and standard deviation close to one. In the end, five representative k-shape clusters were identified and used to quantify transient ICP elevations. The increase in the ICP was calculated as the difference between the lowest and highest ICP values during a transient ICP elevation, and only transient ICP elevations above 6 mmHg were included in the analysis. Each patient’s average ICP value during sleep was calculated from the sleep onset to sleep offset in the hypnogram (including positional change, apneas, and short awake periods).

### The relationship between transient ICP elevations and sleep-disordered breathing

To determine the relationship between sleep apnea and transient ICP elevations, we measured the time difference between the onset of all apneas and the local minima (lowest value) of a transient ICP elevation detected by the automatic macropattern identification method.

### ICP changes during sleep-disordered breathing events

We executed a systematic approach to process alterations in ICP during apnea events across sleep stages. To reduce signal noise while preserving significant trends, the ICP signal was smoothed using a Savitzky‒Golay filter, employing a window size of 257 (corresponding to 1 s) and a second-degree polynomial. Subsequently, we identified the sleep stage corresponding to each apnea event, and the cumulative count of apnea events occurring in each sleep stage. For each isolated apnea event, the magnitude of ICP change was quantified as the difference between the maximum and minimum ICP recorded during the event. We computed the mean and standard deviation of all ICP changes for each patient across the recorded events. To perform a comparative analysis of ICP changes across all apnea events for all patients, the ICP data for all the events were normalized by subtracting the initial ICP value recorded at the onset of an event from all successive readings within the same event, thereby accounting for the inherent variations in absolute ICP values between patients and over time. To determine the standard error of the mean (SEM) at each time point for the compiled dataset comprising all apnea events across the patients. The standard deviation of the ICP changes for each event was divided by the square root of the number of events for each time point across all patients.

### Statistical analysis

Descriptive statistics were obtained for each variable. Summary statistics included the continuous variables’ mean, median, range, standard deviation (SD), and standard error of the mean (SEM). Percentages and sample sizes were used to summarize categorical variables. One-way analysis of variance (ANOVA) was used to compare the groups’ means for continuous variables. Linear regression was applied to test for the association of two independent variables. Values of *P* < 0.05 were considered statistically significant.

### Data availability statement

Anonymized data are available upon reasonable request from the corresponding author and after clearance by the competent ethics committee.

## Results

### Transient ICP elevations

**Fig. 1:**
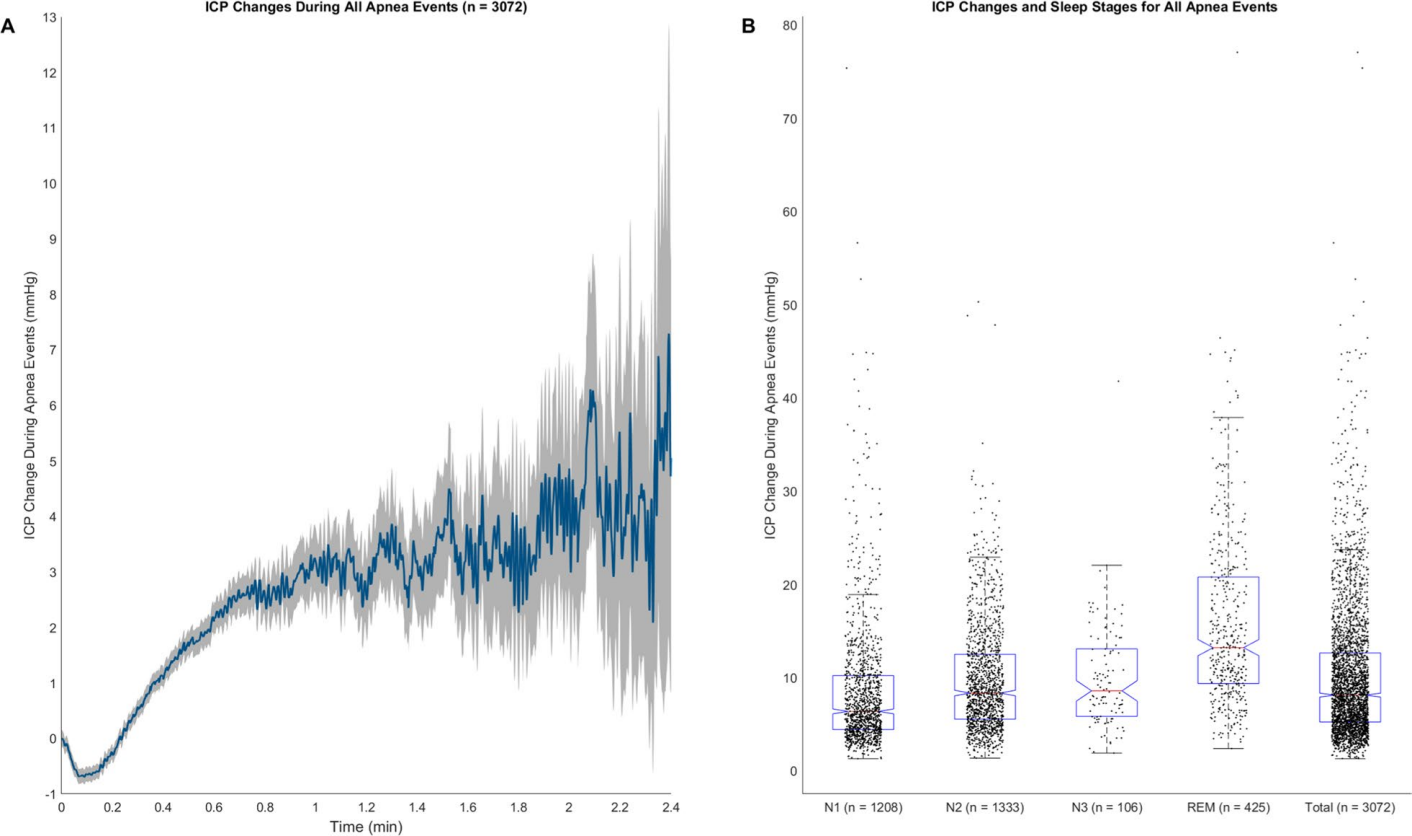
Visualization of ICP changes during all apnea events across all patients. **A** The average ICP change during apnea events is plotted over time. The shaded region indicates the SEM at each time point for the compiled dataset comprising all apnea events across the patients. **B** Boxplots demonstrating the distribution of ICP changes across the sleep stages (N1, N2, N3, REM) and the total, along with individual data points. Each box indicates the interquartile range (IQR), with the median ICP change highlighted by a red line. The whiskers extend from the box to data points within 1.5 times the IQR. Median ICP change for N1: 6.4 mmHg, N2: 8.4 mmHg, N3: 8.6 mmHg, REM: 13.2 mmHg, and total: 8.1 mmHg

**Fig. 2:**
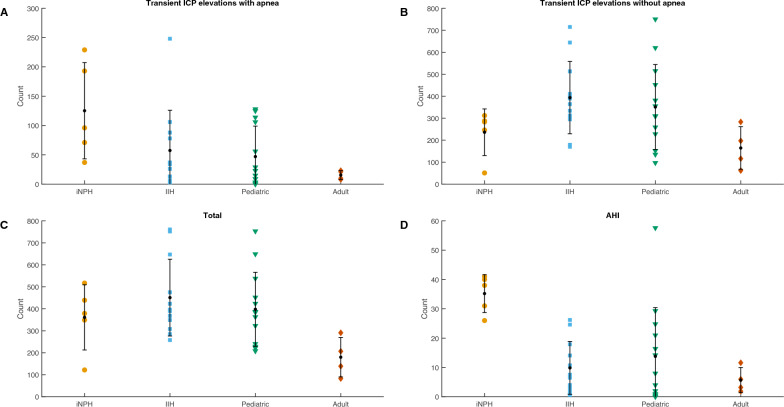
Distribution of individual data points of transient ICP elevations identified by the automatic macropattern algorithm. Transient ICP elevations associated with sleep apnea (**A**), transient ICP elevations not associated with sleep apnea (**B**), the total number of transient ICP elevations identified (**C**), and the apnea–hypopnea index (AHI) for each patient (**D**). Each whisker indicates the standard deviation (SD), with the mean value as a dot (mean and SD are summarized in s Additional file 1: Table S2)

**Fig. 3:**
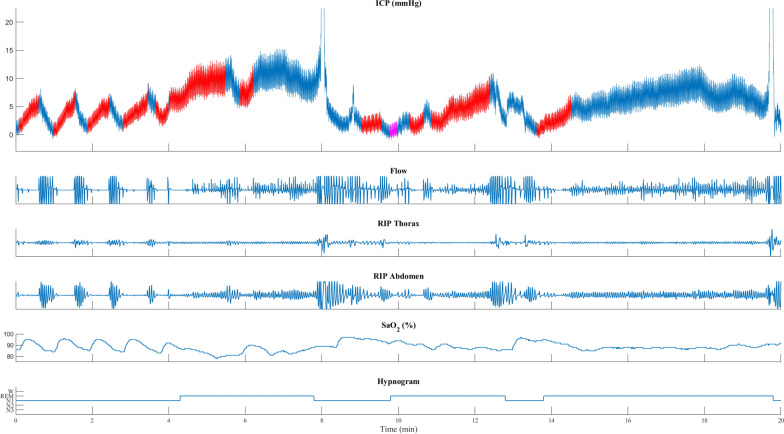
Patient with iNPH with the transition from NREM to REM sleep. The peak in the transient ICP elevations is when respiration resumes, after an apnea, with a ventilatory overshoot marked by increased flow and respiratory movements in the thorax and abdominal RIP. In NREM, in the beginning, repeated transient ICP elevations with a low increase in ICP are seen together with desaturations. During the transition to REM sleep, the transient ICP elevation increases with more significant desaturations and more prolonged apneas. ICP is shown in blue, with red and purple indicating the duration of apneas and respiratory disturbances. Flow: nasal cannula registering flow changes (arbitrary units). RIP, respiratory inductance plethysmography; thorax and abdomen movements (arbitrary units). SaO_2_ (%), oxyhemoglobin saturation measured on the finger. Heart rate, beats/min. Hypnogram with awake and sleep stages

**Fig. 4:**
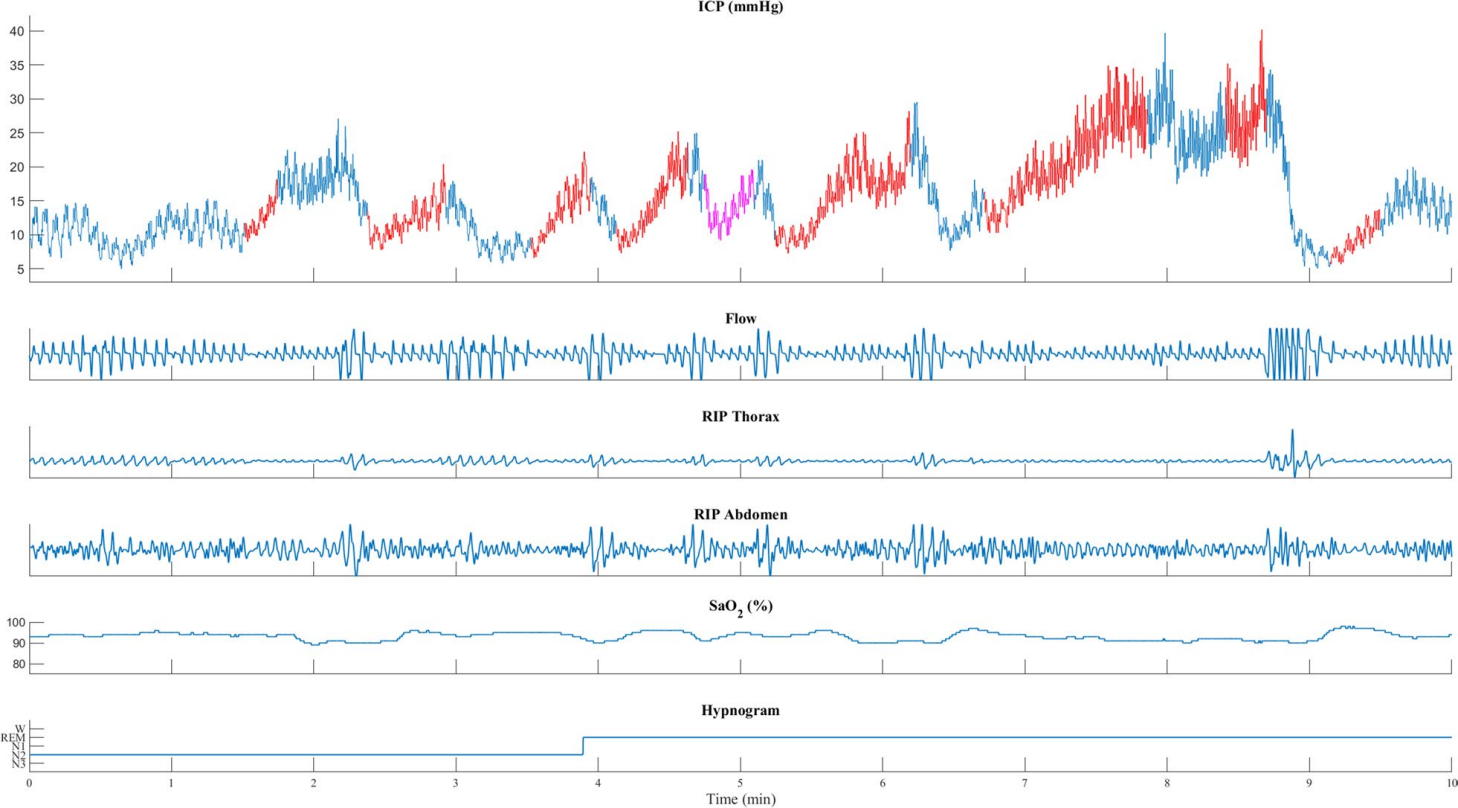
Patient with IIH transitioning from NREM to REM sleep. ICP elevations with sleep apnea start when transitioning from NREM to REM. More activity was observed in abdominal RIP than in thoracic RIP. ICP is shown in blue, with red and purple indicating the duration of apneas and respiratory disturbances. Flow: nasal cannula registering flow changes (arbitrary units). RIP, respiratory inductance plethysmography; thorax and abdomen movements (arbitrary units). SaO_2_ (%), oxyhemoglobin saturation measured on the finger. Heart rate, beats/min. Hypnogram with awake and sleep stages

**Fig. 5:**
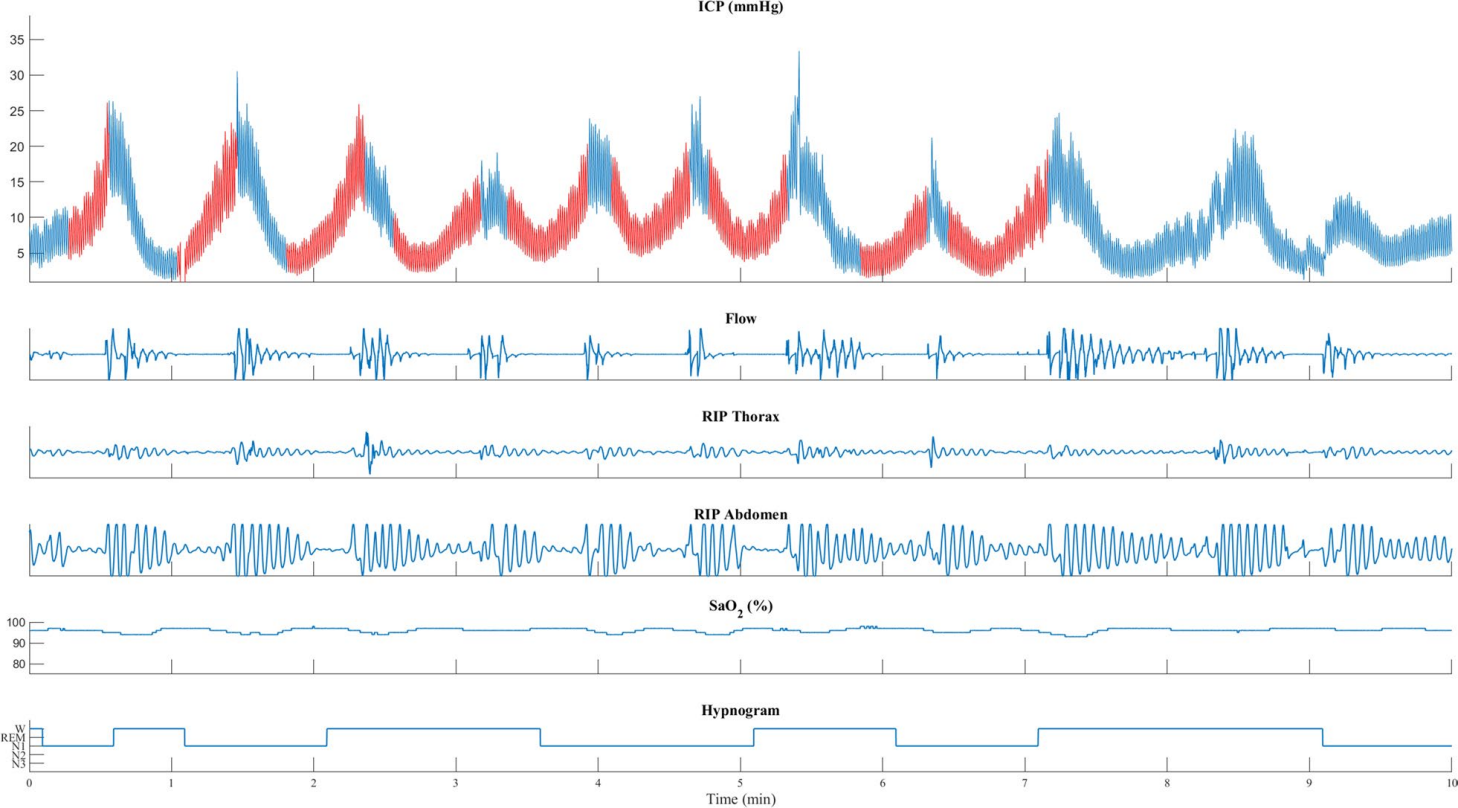
Patient with iNPH and SLEEP APNEA during NREM with frequent arousals and awakenings. The peak of the transient ICP elevation is seen when respiration resumes with a ventilatory overshoot, marked by increased flow and respiratory movements in the thorax and abdominal RIP. All transient ICP elevations in this example are seen with stable oxygen saturation. ICP is shown in blue, with red and purple indicating the duration of apneas and respiratory disturbances. Flow: nasal cannula registering flow changes (arbitrary units). RIP, respiratory inductance plethysmography; thorax and abdomen movements (arbitrary units). SaO_2_ (%), oxyhemoglobin saturation measured on the finger. Heart rate, beats/min. Hypnogram with awake and sleep stages

In our patient cohort, every patient exhibited transient ICP elevations during sleep apnea events (Figs. 1A, 2, 3, 4, and 5). These elevations consistently displayed a distinct pattern, featuring an initial decrease in ICP followed by a subsequent increase and significant fluctuations towards the end of the apnea events. This pattern is exemplified in Fig. 1A, displaying the average transient ICP change across all patients and all apnea events (n = 3072). The results further showed that all types of events, including hypopneas, obstructive events, and respiratory arousals, followed a similar pattern of initial ICP decrease followed by an increase. However, the magnitude of the ICP increase varied across event types, with the highest seen in hypopneas and obstructive events, and the lowest during respiratory arousal events (Additional file 1: Fig. S1–S5).

On average, there was a temporal delay of 3.6 s (SEM 0.2s) noted in 3273 ICP elevations detected by the automatic macropattern identification in the ICP signal between the onset of sleep apnea and the transient ICP elevations, with no significant differences across the groups (*P* = 0.59). Thus, on average, apnea started 3.6 s before the transient ICP elevation, proceeded through most of the upslope of the ICP elevation, and terminated just before the peak of the ICP elevation.

Transient ICP elevations during REM sleep were often associated with larger ICP increases exhibiting ramp-type morphology (Figs. 1B, 3 and 4), but similar changes were also seen during NREM sleep (Fig. 5). In contrast, the ICP elevations observed during NREM sleep often demonstrated a sinusoidal pattern, characterized by smaller increases. For instance, the mean ICP change for REM sleep was 15.8 mmHg and the median ICP change was 13.2 mmHg. By comparison, other sleep stages such as N1, N2, and N3 exhibited lower mean and median ICP changes (Fig. 1B). The highest ICP changes were seen in hypopnea and obstructive events, especially during REM sleep. On the other hand, respiratory arousal events and central events were associated with comparatively smaller ICP changes (Additional file 1: Fig. S1–S5).

All patients had transient ICP elevations not associated with sleep apnea. However, most of these transient ICP elevations had changes in the respiratory signals not meeting the criteria for apneas or hypopneas; thus, in all patients, transient ICP elevation was seen without apneas, most often caused by irregular breathing with altered thoracic and abdominal movement (Additional file 1: Fig. S6).

The number of ICP elevations associated with sleep apnea out of the total number of transient ICP elevations varied between the patient groups. The percentage reflects the mean AHI found in each patient group (Fig. 2 and Additional file 1: Table S2).

### REM sleep ICP elevations

In one patient with iNPH, one with pediatric-onset hydrocephalus, and one with adult-onset hydrocephalus, plateau ICP wave morphology was observed with a prolonged desaturation at the onset of REM sleep periods (Fig. 3).

In eight out of 12 patients with IIH and eight out of 13 patients with pediatric-onset hydrocephalus, REM sleep onset was associated with marked ICP elevations, standing out from the ICP measurement (Additional file 1: Fig. S7). REM sleep ICP elevations followed the same pattern in all these patients, with the highest ICP values at REM sleep onset followed by a declining trend and ending with a decrease when the REM sleep ended (Additional file 1: Fig. S8). This pattern was not observed in patients with iNPH or adult-onset obstructive hydrocephalus.

One patient with IIH and four out of 13 patients with pediatric-onset hydrocephalus had transient ICP elevations not associated with apneas or irregular breathing with altered thoracic and abdominal movement, predominantly during REM sleep and without any change in O_2_ saturation, which was constant at 98–99% (Additional file 1: Fig. S9).

### CO_2_

Two patients with IIH and two with pediatric-onset hydrocephalus measured with end-tidal CO_2_ had nonoscillating CO_2_ levels within normal limits (35–45 mmHg) during their transient ICP elevations (Additional file 1: Fig. S10).

### ICP

The average ICP was highest in the patients with IIH [15.1 (6.6)], with only one patient having an average ICP below 10 mmHg (7.3 mmHg). The average ICP was 10.4 (6.7) for the pediatric-onset patients, 7.2 (2.3) for the adult-onset patients, and 6.7 (1.9) for the patients with iNPH (ANOVA*, P* = 0.036). The post hoc confirmatory t-test showed the only significant difference between patients with iNPH and IIH (*P* = 0.0014) and adult-onset and IIH (*P* = 0.0035). The average duration of the ICP measurements included in the analysis was 520 (147) minutes. Linear regression showed no association between the total number of ICP elevations and the average ICP for any patient group (*R*^*2*^ = − 0.019, *P* = 0.54). However, as expected, there was a significant association between the mean increase in the ICP during the transient ICP elevations and the average ICP (*R*^*2*^ = 0.32, *P* = 0.00031). All data are shown in Additional file 1: Table S1.

### CPAP

Three patients were monitored during an additional night with CPAP treatment (two patients with iNPH, one with pediatric-onset hydrocephalus, and a ventriculoperitoneal (VP)-shunt). All three patients used CPAP treatment most of the night, and all were above the compliance threshold of > 4 h per night. Furthermore, they all had a low level of leakage, indicating a good mask fit, and the mean pressure applied by CPAP was within normal limits (Additional file 1: Table S3).

The mean reduction of transient ICP elevations in all three patients with CPAP treatment was 37% (Table 2). There was an apparent reduction in transient ICP elevations with and without apneas for one of the patients with iNPH and the pediatric-onset patient. However, the second patient with iNPH had predominantly central apneas unaffected by CPAP. Accordingly, there was no reduction in transient ICP elevations associated with apneas but only in transient ICP elevations without apneas. CPAP treatment resulted in insignificant changes in the average ICP in two patients but elevated the average ICP during sleep in one patient by 5.6 mmHg (Additional file 1: Table S2).

**Table 2:** Quantification of transient ICP elevations in three patients measured with and without continuous positive airway pressure (CPAP)

	First night			Second night with CPAP			Change (%)
	Total	Apnea	without apnea	Total	Apnea	without apnea	
iNPH 1	439	193	246	210	38	172	− 52.2
iNPH 2	379	96	283	223	105	118	− 41.2
Pediatric	241	106	135	200	1	199	− 17.0
Mean	353	132	221	211	48	163	− 37.0

The change indicates the reduction in the total number of transients ICP elevation with CPAP treatment

*iNPH* Idiopathic normal pressure hydrocephalus; *Pediatric* pediatric-onset hydrocephalus

## Discussion

Our main finding is that transient ICP elevations are associated with sleep apnea in all patients in this study. Transient ICP elevations with a larger increase in ICP and ramp-type transient ICP elevations were related to sleep apnea during REM sleep and a lower ICP increase was related to sinusoidal-type with NREM sleep. All patients had transient ICP elevations without sleep apnea, especially those with IIH and pediatric-onset hydrocephalus, with similar ICP changes during REM sleep periods. Furthermore, CPAP treatment reduced the number of transient ICP elevations and changed the average ICP during sleep.

### Influence of sleep-disordered breathing on transient ICP elevations

ICP is influenced by various factors, including the volume of intracranial components (e.g., brain tissue, blood, CSF), the compliance of the intracranial cavity, and the resistance to cerebrospinal fluid outflow. Notably, sleep apnea is associated with several physiological changes that could impact ICP, such as alterations in heart rate, blood pressure, and respiratory function. Interestingly, our findings indicate that sleep apnea triggers transient ICP elevations in all participants, as shown previously in patients without ICP disturbances [4]. This suggests that transient ICP elevations may be a common physiological consequence of sleep apnea.

Transient ICP increases do not occur with regular intervals or durations. They have very different appearances and durations between patients and within patients, which speaks against a universal underlying and generating mechanism. This favors a role for sleep apnea in generating transient ICP elevations in many of the observed ICP elevations, as apneas and hypopneas vary in duration, degree, and timing. The timing of the apnea in the respiratory cycle, the level of negative intrathoracic pleural pressure, or the apnea duration could explain some of the heterogeneity in the sleep apnea generated transient ICP elevations we observed since short apneas and irregular breathing only caused transient ICP elevation with a minor increase in the ICP (Additional file 1: Fig. S5). However, several mechanisms are probably responsible for generating transient ICP elevations.

During hypopneas and obstructive events, partial or complete upper airway obstruction reduces airflow. This obstruction results in increased breathing effort, increasing negative intrathoracic pleural pressure. The increased effort to overcome the obstruction causes the diaphragm and other respiratory muscles to contract more forcefully, generating a more significant negative pressure in the thoracic cavity. On the other hand, respiratory arousals and central events do not involve upper airway obstruction. Respiratory arousals are brief awakenings from sleep due to changes in respiratory drive or increased respiratory effort, while central events occur when respiratory effort temporarily ceases. These events do not typically involve the same level of an increased effort to breathe as seen in hypopneas and obstructive events. As a result, the negative intrathoracic pleural pressure generated during respiratory arousals and central events is generally lower than that generated during hypopneas and obstructive events. Following this, the most significant ICP changes were seen in hypopnea and obstructive events, especially during REM sleep, and respiratory arousal events and central events were associated with comparatively more minor ICP changes (Additional file 1: Fig. S1–S5), suggesting that the level of negative intrathoracic pleural pressure is important for ICP changes.

### CO_2_ and transient ICP elevations

Traditionally, CO_2_ has been considered essential in generating transient ICP elevations, but several studies have shown conflicting results [1, 4, 17–19]. Recently, we have shown that transient ICP elevations (B-waves) can be generated by sleep apnea and abnormal respiratory movements in the chest and not by elevated levels of PaCO_2_ when ICP is below 20 mmHg [4]. Cerebral blood flow (CBF) regulation, through vasodilation or constriction of the cerebral blood vessels, is critical for the cerebrovasculature to respond to changes in O_2_ and CO_2_, resulting in changes in cerebral blood volume (CBV) and ICP [20–22].

Furthermore, ventilation and PaCO_2_ are closely linked. Thus, CO_2_ is critical for CBF regulation and stable breathing, and compromised regulation of CBF can lead to irregular breathing. Accordingly, CO_2_ is most likely a modulator of transient ICP elevations when ICP is within normal levels and may primarily affect transient ICP elevations by influencing the rhythm of breathing and secondary in changing the arterial CBV. Accordingly, our results show sleep apnea-associated transient ICP elevations without changes in CO_2_ or O_2_ (Fig. 5 and Additional file 1: Fig. S10). However, increased CO_2_ is probably an important factor in the transient ICP elevations with plateau morphology seen in the three patients with REM sleep prolonged desaturation.

### Physiology of transient ICP elevations

The total CBV consists of 70% capillary and venous blood and only 30% arterial blood [23], and changes in CBV during hypercapnia and hypocapnia only cause changes in arterial blood volume without changes in venous and capillary blood volume [24]. Thus, rapid changes in the outflow of venous blood, not affected by CO_2_, have significant potential to alter ICP.

Respiratory inhalation drives deoxygenated venous blood outflow from the brain and induces a counterbalancing of CSF inflow, following the Monro–Kellie doctrine of relative compartment changes [25, 26]. A temporary cessation of breathing, as during apnea, will generate negative intrathoracic pleural pressure increasing venous return and initially decreasing ICP. However, as apnea precedes, an excessive venous return to the heart will raise the central venous pressure (CVP) [5] and ultimately decrease cerebral venous blood outflow, increasing CVP and ICP. When apnea stops and breathing is restored, the intrapleural pressure will change to positive instantly, thus further increasing CVP and ICP. Our results are consistent with this physiological association between sleep apnea and transient ICP elevations, as there is a close temporal delay between the onset of apnea and the subsequent increase in ICP. On average, in a total apnea count of 3270 in all patients, apnea started 3.6 s before the lowest ICP level and proceeded throughout the ICP elevation ending close to the peak of the ICP increase.

Similarly, the average ICP during all apnea events across all patients (Fig. 1A) showed an initial drop in ICP, followed by a steady increase in ICP during the apnea event. The ICP peak coincides with a ventilatory overshoot when respiration resumes (Figs. 3, 4, and 5). These findings are supported by a close-phase correlation of transient ICP elevations with the peak of respiratory movements, CVP, and arterial blood pressure, with or without changes in CO_2_ [5, 19, 27].

### Transient ICP elevations without sleep-disordered breathing

During the REM sleep phase, ICP elevations in patients with IIH and pediatric-onset hydrocephalus were also observed without changes in respiration and oxygen saturation or CO_2_ (Additional file 1: Fig. S9). This suggests that other mechanisms generate transient ICP elevations and that the mechanism may differ depending on the underlying clinical condition. In particular, patients with IIH and pediatric-onset hydrocephalus display similar ICP morphology compared to patients with iNPH and adult-onset hydrocephalus.

**Fig. 6:**
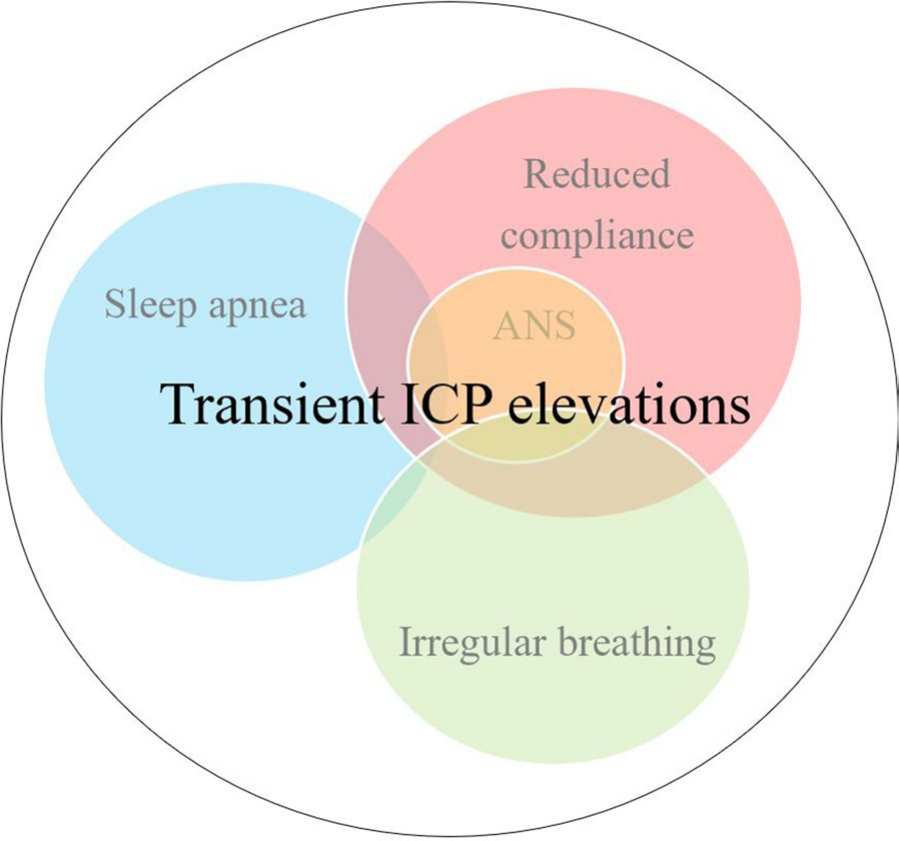
Different mechanisms generate transient ICP elevations. Sleep apnea generates a significant proportion of transient ICP elevations, followed by irregular breathing with altered thoracic and abdominal movement. However, during reduced compliance, often seen by pathologically high ICP > 20 mmHg, transient ICP elevations are generated by slow oscillating changes in the autonomic system (ANS) reflected in the cardiovascular system

The increase in ICP during REM sleep onset is likely due to changes in CBF, which is known to increase during REM sleep rhythmically [28–30]. However, it is possible that CSF volume changes are also involved, although CSF is more likely to be involved in the slowly declining ICP level during the REM sleep phase. This decline may be due to a slowly adapting CSF circulation, where CSF is drained from the ventricles into the spinal canal. Physiological alterations occurring during REM sleep could be critical in generating transient ICP elevations, especially in cases where ICP is pathologically elevated. Thus, we speculate that different underlying mechanisms cause transient ICP elevations during REM sleep but are often caused by sleep apnea or changes in respiration. However, in some patients, especially during high ICP or reduced venous buffer capacity due to venous hypertension, transient ICP elevations may be generated by oscillating changes in the cardiovascular system dictated by the autonomic nervous system. Future studies should try to pinpoint the mechanism generating these slow oscillations. Figure 6 summarizes our hypothesis regarding the physiology of transient ICP elevations during sleep.

### Clinical implications

#### iNPH and sleep-disordered breathing

The link between sleep apnea and patients with ICP changes has been known for many years [1, 5–7], and patients with iNPH are particularly prone to severe sleep apnea [8–10], which has also been associated with cerebral microbleeds and brain ischemias. As shown in Fig. 2 and Additional file 1: Table S2, patients with iNPH had the highest number of sleep apnea-associated transient ICP elevations among the four groups of patients. Transient increases in ICP caused by sleep apnea, as shown here and previously [4, 5, 31], could be involved in the progression of chronic hydrocephalus, particularly in patients with iNPH, and require further investigation. It is also worth considering whether transient ICP elevations generated by sleep apnea could play a role in the well-known association between sleep apnea, stroke, and cardiovascular disease [32]. An animal model of sleep apnea with simultaneous ICP measurement could also help elucidate the mechanism of sleep apnea in generating transient ICP elevations.

#### CPAP

In three treated patients, CPAP reduced the number of transient increases in ICP. However, in one patient with iNPH, the average ICP increased, possibly due to CPAP causing increased resistance to venous return, as reported in previous studies [33, 34]. It is also possible that this was a general consequence of CPAP treatment. A case report documented worsening symptoms in a patient with iNPH after starting CPAP treatment but showed improvement after undergoing a VP-shunt procedure [35]. Patients with iNPH have a higher frequency of retrograde jugular venous flow, possibly because of incompetent jugular valves. This may also contribute to the observed increase in ICP during CPAP treatment [36]. Future studies should measure both ICP and pressure in the venous system during CPAP treatment to understand the impact of CPAP on ICP and venous pressure in patients.

Interestingly, in the patient with pediatric-onset hydrocephalus and a VP-shunt, CPAP treatment appeared to reduce the average ICP, but the decrease was only 1 mmHg and insignificant. (Additional file 1: Table S3). Future studies should elucidate the effects of CPAP on ICP, particularly in patients with hydrocephalus, as CPAP could potentially be harmful if it increases ICP.

## Limitations

The primary limitation of this study is the relatively small sample size (*n* = 34). However, even though the sample size was small, the results are consistent within each patient group regarding the association of sleep apnea with transient ICP elevations. Despite the relatively limited sample size in this study, it represents the most comprehensive research of its kind ever undertaken.

We used an objective method to detect the number of transient ICP elevations. Thus, we most likely overestimated the number of transient ICP elevations in some patients since regular small movements and positional changes are reflected in the ICP signal and count as a transient ICP elevation. However, the objective method used in this study reduces bias and makes the data more reproducible. Others have used objective methods to calculate transient ICP elevations and found an association between transient ICP elevations and the average ICP [11]. In contrast, we found no association between transient ICP elevations and the average ICP but only, as expected, an association between the average increase in the ICP during the transient ICP elevations and the average ICP. The algorithm and the threshold used could explain differences since they used a 0.5 to 5.0 mmHg threshold. This highlights the importance of selecting a proper threshold for detecting clinically relevant transient ICP elevations, which probably should be > 5.0 mmHg.

A telemetric ICP probe was used in one patient with pediatric-onset hydrocephalus and six with IIH (Additional file 1: Table S1). Zero-offset drift is a potential source of error in the telemetric probe and affects the average ICP results. However, recognizable morphological changes are still detectable in the signal, such as transient ICP elevations [4]. There were no technical differences in the ICP signal in patients with telemetric probes compared to the remaining patients. Fourteen patients were measured with a parenchymal ICP probe, with the same high sampling rate and no zero-offset drift as the gold standard ventricular probe used in the remaining patients (Additional file 1: Table S1). Thus, minor deviations in absolute ICP may occur depending on the tip location of the pressure gauge.

Some patients underwent treatment involving a VP-shunt or ETV, potentially impacting the measured average ICP (Additional file 1: Table S1). Notably, prior research by us and others has demonstrated that VP-shunt treatment does not change the prevalence of sleep apnea among patients with iNPH [8–10]. Importantly, the frequency or occurrence of transient ICP elevations remains unaffected by these treatments. However, alterations in ICP during such episodes could potentially be influenced by the presence of a VP-shunt or an ETV. Thus, VP-shunt or ETV treatment interventions might modify the absolute ICP changes while not influencing the frequency or occurrence of sleep apnea or transient ICP elevations.

Nonetheless, it is pertinent to acknowledge the possibility that treatment with a VP-shunt or ETV may impact the broader macro pattern of ICP, particularly during phases such as REM sleep.

## Conclusion

The findings suggest that sleep apnea causes a significant proportion of transient ICP elevations, such as B-waves, and sleep apnea should be considered in ICP evaluation. Treatment of sleep apnea with CPAP can reduce the occurrence of transient ICP elevations. More research is needed on the impact of slow oscillating mechanisms on transient ICP elevations during high ICP and REM sleep.

## Supplementary Information

**Additional file 1: Figure S1.** Visualization of ICP changes during all obstructive events across all patients. **A** The average ICP change during apnea events is plotted over time. The shaded region indicates the SEM at each time point for the compiled dataset comprising all obstructive events across the patients. **B** Boxplots demonstrating the distribution of ICP changes across the sleep stages (N1, N2, N3, REM) and the total, along with individual data points. Each box indicates the interquartile range (IQR), with the median ICP change highlighted by a red line. The whiskers extend from the box to data points within 1.5 times the IQR. **Figure S2**. Visualization of ICP changes during all hypopnea events across all patients. **A** The average ICP change during apnea events is plotted over time. The shaded region indicates the SEM at each time point for the compiled dataset comprising all hypopnea events across the patients. **B** Boxplots demonstrating the distribution of ICP changes across the sleep stages (N1, N2, N3, REM) and the total, along with individual data points. Each box indicates the interquartile range (IQR), with the median ICP change highlighted by a red line. The whiskers extend from the box to data points within 1.5 times the IQR. **Figure S3.** Visualization of ICP changes during all central events across all patients. **A** The average ICP change during apnea events is plotted over time. The shaded region indicates the SEM at each time point for the compiled dataset comprising all central events across the patients. **B** Boxplots demonstrating the distribution of ICP changes across the sleep stages (N1, N2, N3, REM) and the total, along with individual data points. Each box indicates the interquartile range (IQR), with the median ICP change highlighted by a red line. The whiskers extend from the box to data points within 1.5 times the IQR. **Figure S4.** Visualization of ICP changes during all mixed events across all patients. **A** The average ICP change during apnea events is plotted over time. The shaded region indicates the SEM at each time point for the compiled dataset comprising all mixed events across the patients. **B** Boxplots demonstrating the distribution of ICP changes across the sleep stages (N1, N2, N3, REM) and the total, along with individual data points. Each box indicates the interquartile range (IQR), with the median ICP change highlighted by a red line. The whiskers extend from the box to data points within 1.5 times the IQR. **Figure S5.** Visualization of ICP changes during all respiratory arousal events across all patients. **A** The average ICP change during apnea events is plotted over time. The shaded region indicates the SEM at each time point for the compiled dataset comprising all hypopnea events across the patients. **B** Boxplots demonstrating the distribution of ICP changes across the sleep stages (N1, N2, N3, REM) and the total, along with individual data points. Each box indicates the interquartile range (IQR), with the median ICP change highlighted by a red line. The whiskers extend from the box to data points within 1.5 times the IQR. **Figure S6.** Pediatric patient with transient ICP elevations with respiratory disturbances in NREM sleep. An increase and decrease are seen in the flow signal and the thorax and abdominal RIP signal with every increase in ICP. ICP is blue, with purple indicating the duration of respiratory disturbances. Flow: nasal cannula registering flow changes (arbitrary units). RIP, respiratory inductance plethysmography; thorax and abdomen movements (arbitrary units). SaO2 (%), oxyhemoglobin saturation measured on the finger. Heart rate, beats/min. Hypnogram with awake and sleep stages. **Figure S7.** NREM to REM transition in a patient with pediatric-onset hydrocephalus. The peak of the transient ICP elevation is seen when respiration resumes with a ventilatory overshoot, marked by increased flow and respiratory movements in the thorax and abdominal RIP. In NREM, the beginning sinusoidal transient ICP elevation with a low ICP increase and frequent arousals is seen with more activity in the thoracic RIP than in the abdominal RIP. ICP is shown in blue, with red and purple indicating the duration of apneas and respiratory disturbances, respectively. Flow: nasal cannula registering flow changes (arbitrary units). RIP, respiratory inductance plethysmography; thorax and abdomen movements (arbitrary units). SaO2 (%), oxyhemoglobin saturation measured on the finger. Heart rate, beats/min. Hypnogram with awake and sleep stages. **Figure S8.** ICP elevations with every REM sleep episode in a patient with IIH. The ICP signal has a declining trend through each REM sleep episode. ICP is blue, with red and purple indicating the duration of apneas and respiratory disturbances. Flow: nasal cannula registering flow changes (arbitrary units). RIP, respiratory inductance plethysmography; thorax and abdomen movements (arbitrary units). SaO2 (%), oxyhemoglobin saturation measured on the finger. Heart rate, beats/min. Hypnogram with awake and sleep stages.** Figure S9.** Patient with IIH and repeating ramp-type transient ICP elevations with the onset of REM without apneas, respiratory disturbances, or desaturation. ICP is shown in blue, with red and purple indicating the duration of apneas and respiratory disturbances. Flow, nasal cannula registering flow changes (arbitrary units). RIP, respiratory inductance plethysmography; thorax and abdomen movements (arbitrary units). SaO2 (%), oxyhemoglobin saturation measured on the finger. Heart rate, beats/min. Hypnogram with awake and sleep stages. **Figure 10.** Repeating ramp-type transient ICP elevations with the onset of REM in a patient with IIH without changes in CO2 and O2. ICP is blue, with red and purple indicating the duration of apnea and respiratory disturbances. Flow: nasal cannula registering flow changes (arbitrary units). RIP, respiratory inductance plethysmography; thorax and abdomen movements (arbitrary units). SaO2 (%), oxyhemoglobin saturation measured on the finger. Heart rate, beats/min. Hypnogram with awake and sleep stages. CO2 was measured with end-tidal LoFLo. **Table S1.** Detailed information about all patients. **Table S2**. Quantification of transient ICP elevations. **Table S3.** Continuous positive airway pressure (CPAP) usage in the three patients.

## Data Availability

Anonymized data are available upon reasonable request from the corresponding author and after clearance by the competent ethics committee.

## Abbreviations

CBF: Cerebral blood flow
CBV: Cerebral blood volume
CPAP: Continuous positive airway pressure
ETV: Endoscopic third ventriculostomy
iNPH: Idiopathic normal pressure hydrocephalus
IIH: Intracranial hypertension
ICP: Intracranial pressure
PSG: Polysomnography
VP-shunt: Ventriculoperitoneal shunt
