# Supplementary material for: Transient intracranial pressure elevations (B waves) are associated with sleep apnea
Source: Fluids Barriers CNS. 2023 Oct 2;20:69. doi: 10.1186/s12987-023-00469-6 (PMC10544378; doi:10.1186/s12987-023-00469-6)
Supplement: Supplementary file 1 — Additional file 1: Figure S1. Visualization of ICP changes during all obstructive events across all patients. A The average ICP change during apnea events is plotted over time. The shaded region indicates the SEM at each time point for the compiled dataset comprising all obstructive events across the patients. B Boxplots demonstrating the distribution of ICP changes across the sleep stages (N1, N2, N3, REM) and the total, along with individual data points. Each box indicates the interquartile range (IQR), with the median ICP change highlighted by a red line. The whiskers extend from the box to data points within 1.5 times the IQR. Figure S2. Visualization of ICP changes during all hypopnea events across all patients. A The average ICP change during apnea events is plotted over time. The shaded region indicates the SEM at each time point for the compiled dataset comprising all hypopnea events across the patients. B Boxplots demonstrating the distribution of ICP changes across the sleep stages (N1, N2, N3, REM) and the total, along with individual data points. Each box indicates the interquartile range (IQR), with the median ICP change highlighted by a red line. The whiskers extend from the box to data points within 1.5 times the IQR. Figure S3. Visualization of ICP changes during all central events across all patients. A The average ICP change during apnea events is plotted over time. The shaded region indicates the SEM at each time point for the compiled dataset comprising all central events across the patients. B Boxplots demonstrating the distribution of ICP changes across the sleep stages (N1, N2, N3, REM) and the total, along with individual data points. Each box indicates the interquartile range (IQR), with the median ICP change highlighted by a red line. The whiskers extend from the box to data points within 1.5 times the IQR. Figure S4. Visualization of ICP changes during all mixed events across all patients. A The average ICP change during apnea events is pl [file 12987_2023_469_MOESM1_ESM.pdf]

Additional file

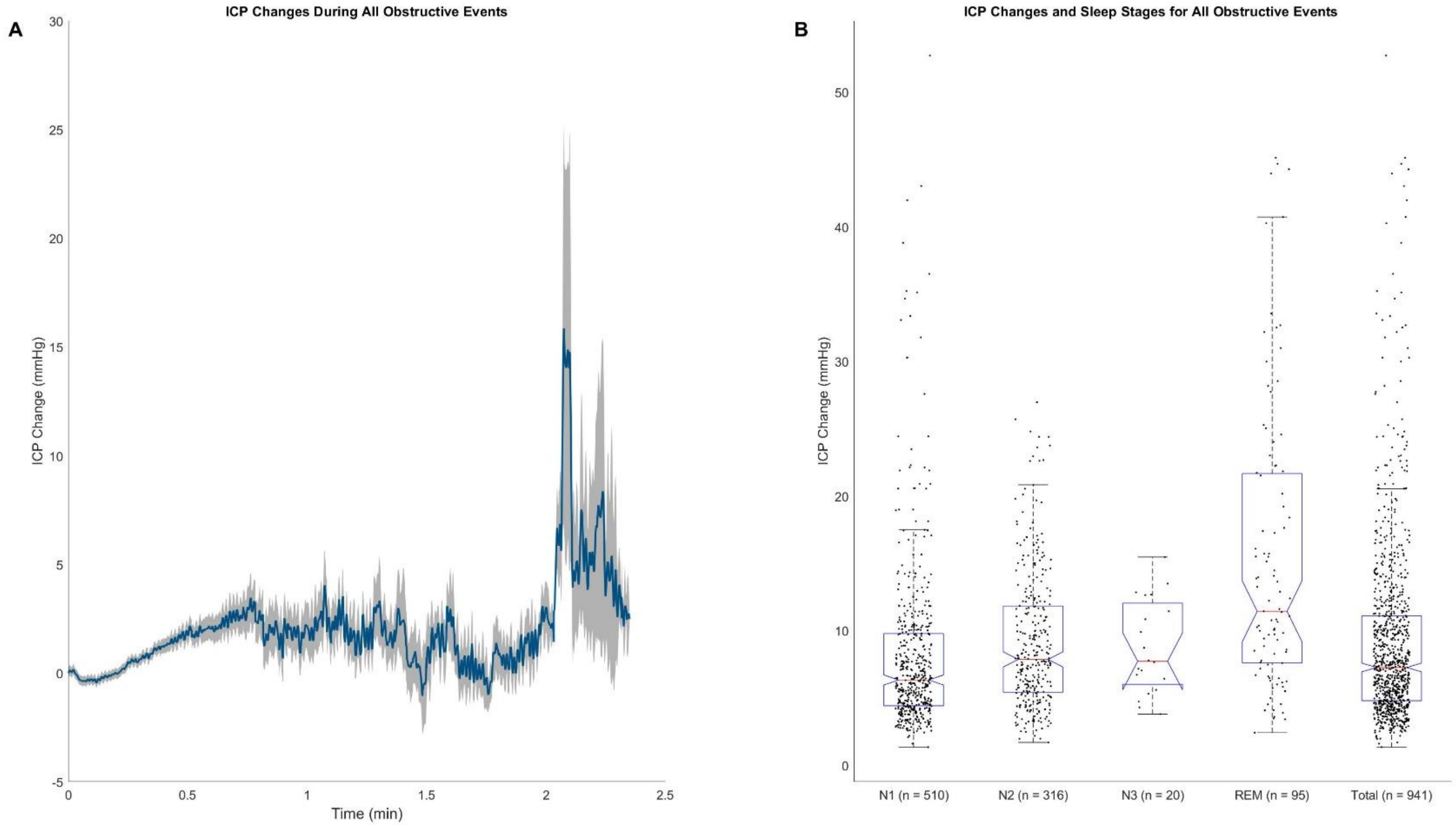

Figure S1: Visualization of ICP changes during all obstructive events across all patients. (A) The average ICP change during apnea events is plotted over time. The shaded region indicates the SEM at each time point for the compiled dataset comprising all obstructive events across the patients. (B) Boxplots demonstrating the distribution of ICP changes across the sleep stages (N1, N2, N3, REM) and the total, along with individual data points. Each box indicates the interquartile range (IQR), with the median ICP change highlighted by a red line. The whiskers extend from the box to data points within 1.5 times the IQR.

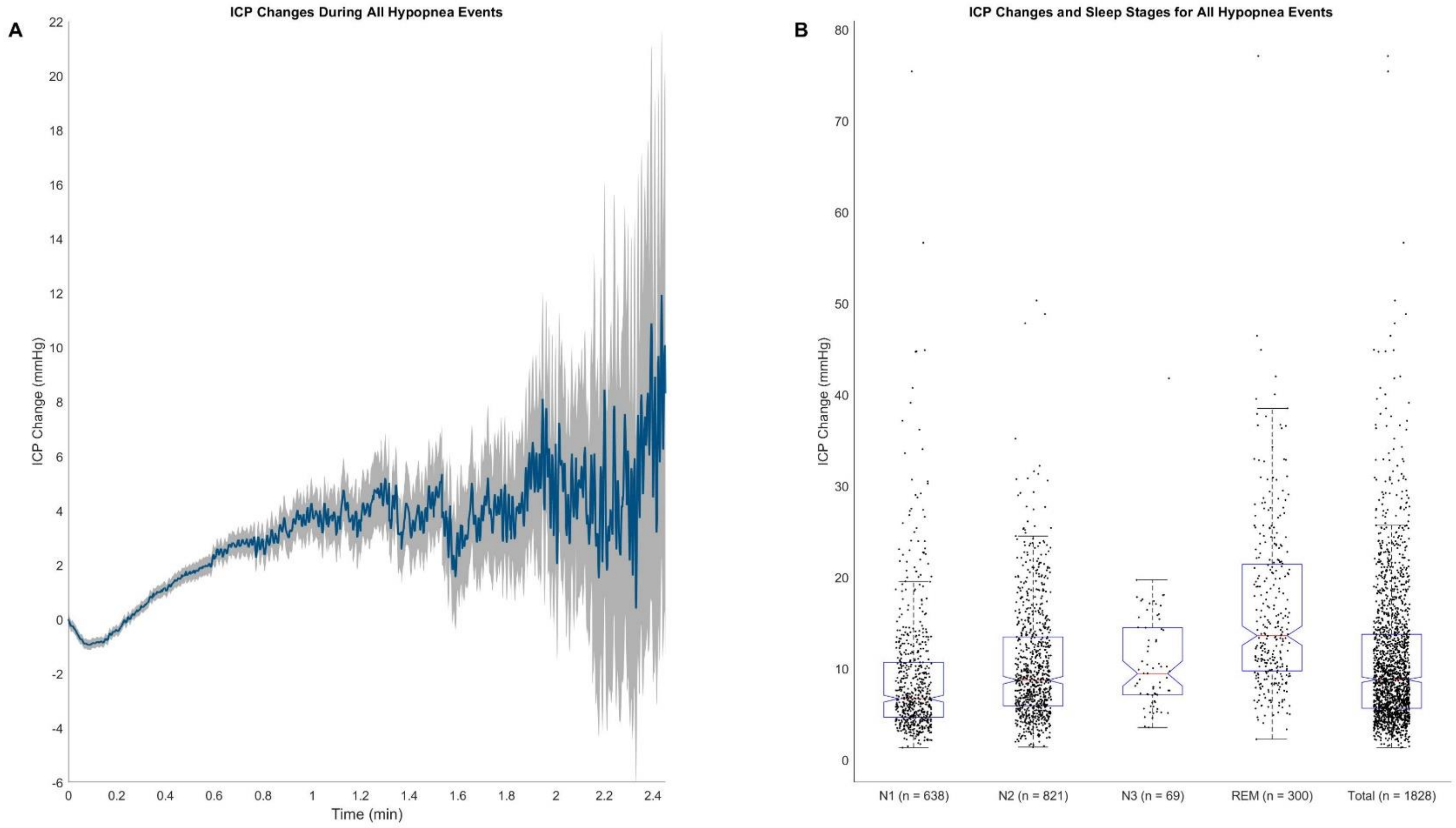

Figure S2: Visualization of ICP changes during all hypopnea events across all patients. (A) The average ICP change during apnea events is plotted over time. The shaded region indicates the SEM at each time point for the compiled dataset comprising all hypopnea events across the patients. (B) Boxplots demonstrating the distribution of ICP changes across the sleep stages (N1, N2, N3, REM) and the total, along with individual data points. Each box indicates the interquartile range (IQR), with the median ICP change highlighted by a red line. The whiskers extend from the box to data points within 1.5 times the IQR.

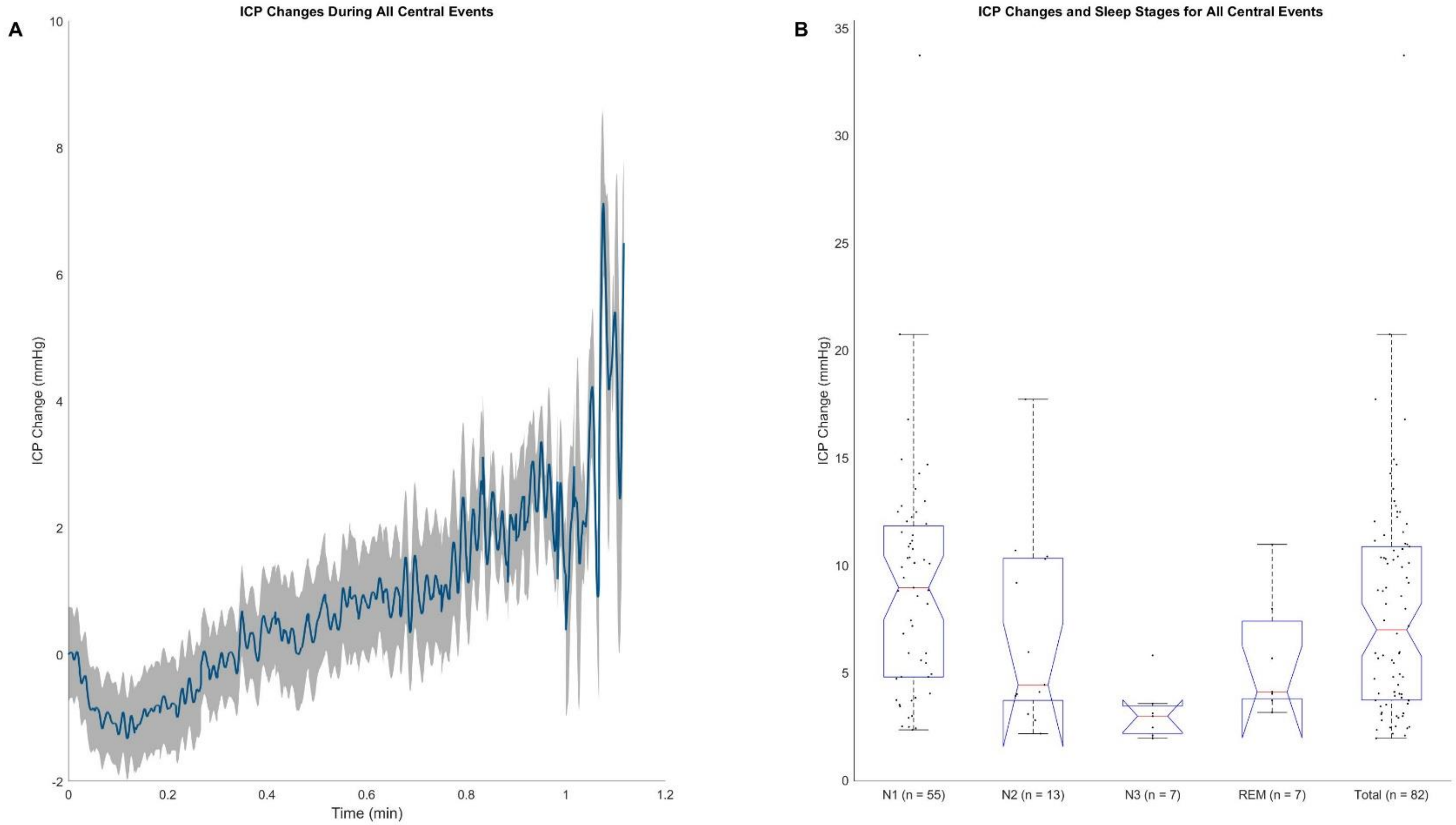

Figure S3: Visualization of ICP changes during all central events across all patients. (A) The average ICP change during apnea events is plotted over time. The shaded region indicates the SEM at each time point for the compiled dataset comprising all central events across the patients. (B) Boxplots demonstrating the distribution of ICP changes across the sleep stages (N1, N2, N3, REM) and the total, along with individual data points. Each box indicates the interquartile range (IQR), with the median ICP change highlighted by a red line. The whiskers extend from the box to data points within 1.5 times the IQR.

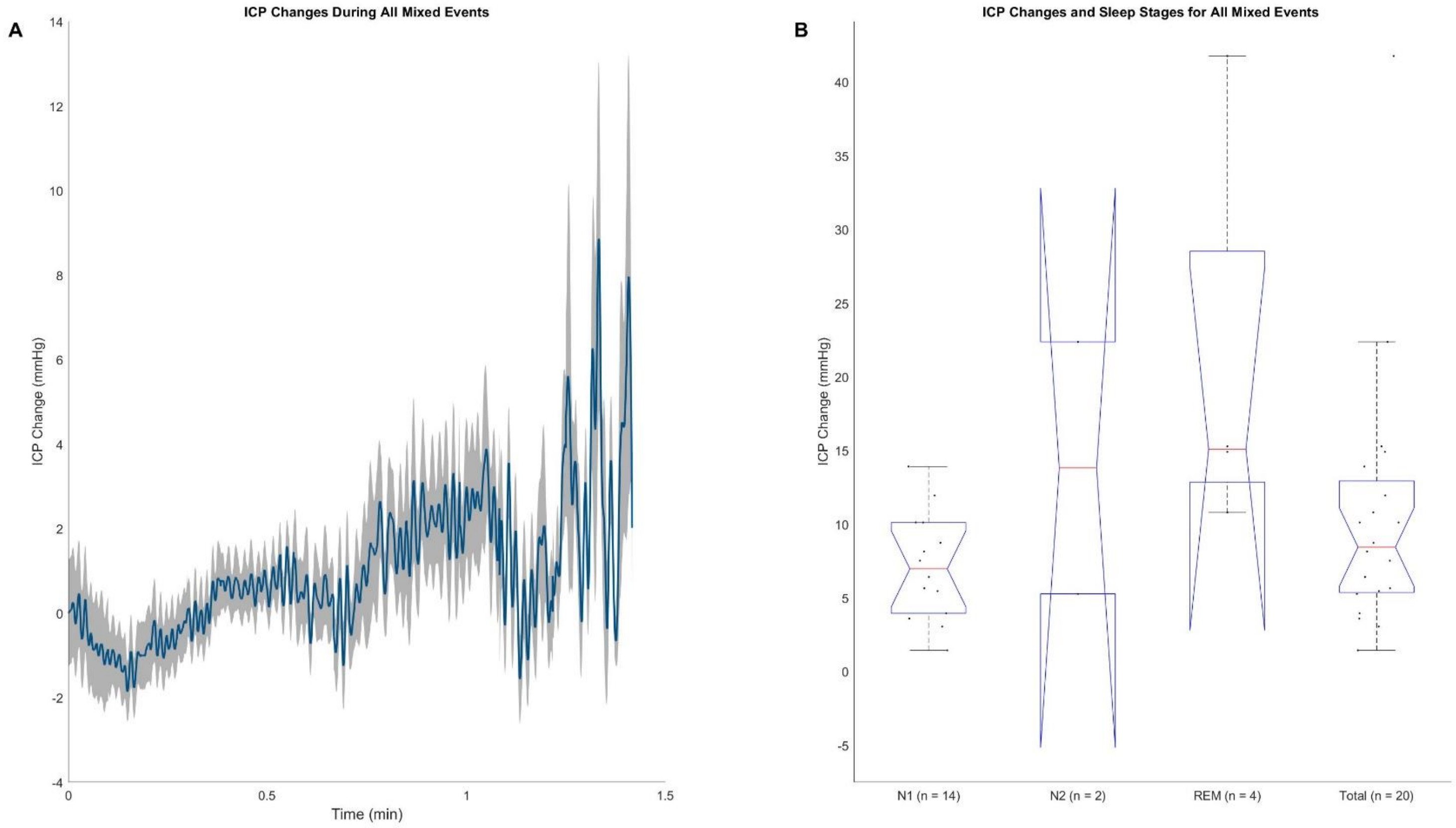

Figure S4: Visualization of ICP changes during all mixed events across all patients. (A) The average ICP change during apnea events is plotted over time. The shaded region indicates the SEM at each time point for the compiled dataset comprising all mixed events across the patients. (B) Boxplots demonstrating the distribution of ICP changes across the sleep stages (N1, N2, N3, REM) and the total, along with individual data points. Each box indicates the interquartile range (IQR), with the median ICP change highlighted by a red line. The whiskers extend from the box to data points within 1.5 times the IQR.

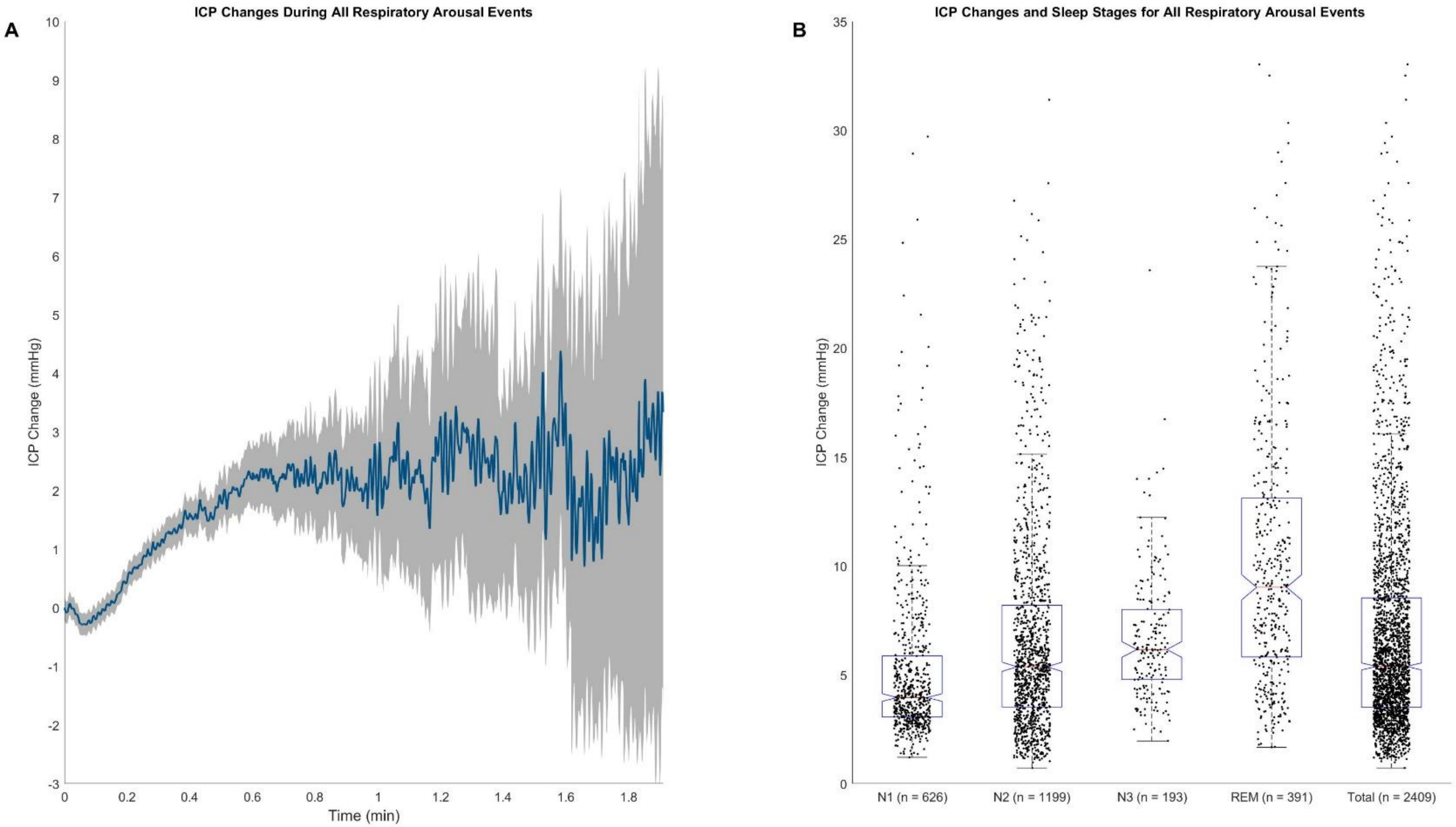

Figure S5: Visualization of ICP changes during all respiratory arousal events across all patients. (A) The average ICP change during apnea events is plotted over time. The shaded region indicates the SEM at each time point for the compiled dataset comprising all hypopnea events across the patients. (B) Boxplots demonstrating the distribution of ICP changes across the sleep stages (N1, N2, N3, REM) and the total, along with individual data points. Each box indicates the interquartile range (IQR), with the median ICP change highlighted by a red line. The whiskers extend from the box to data points within 1.5 times the IQR.

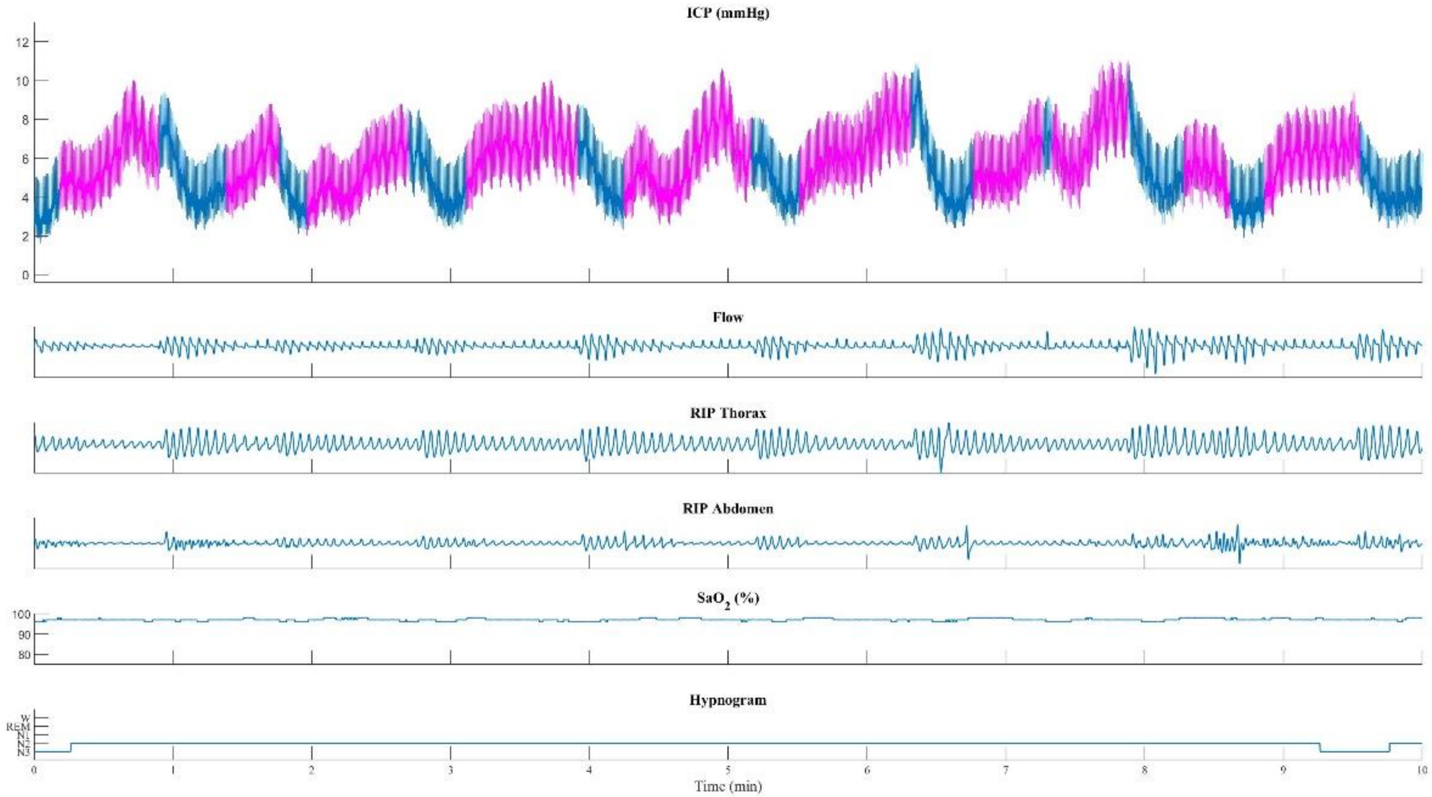

Figure S6 Pediatric patient with transient ICP elevations with respiratory disturbances in NREM sleep. An increase and decrease are seen in the flow signal and the thorax and abdominal RIP signal with every increase in ICP. ICP is blue, with purple indicating the duration of respiratory disturbances. Flow: nasal cannula registering flow changes (arbitrary units). RIP, respiratory inductance plethysmography; thorax and abdomen movements (arbitrary units). SaO_2 (%), oxyhemoglobin saturation measured on the finger. Heart rate, beats/min. Hypnogram with awake and sleep stages.

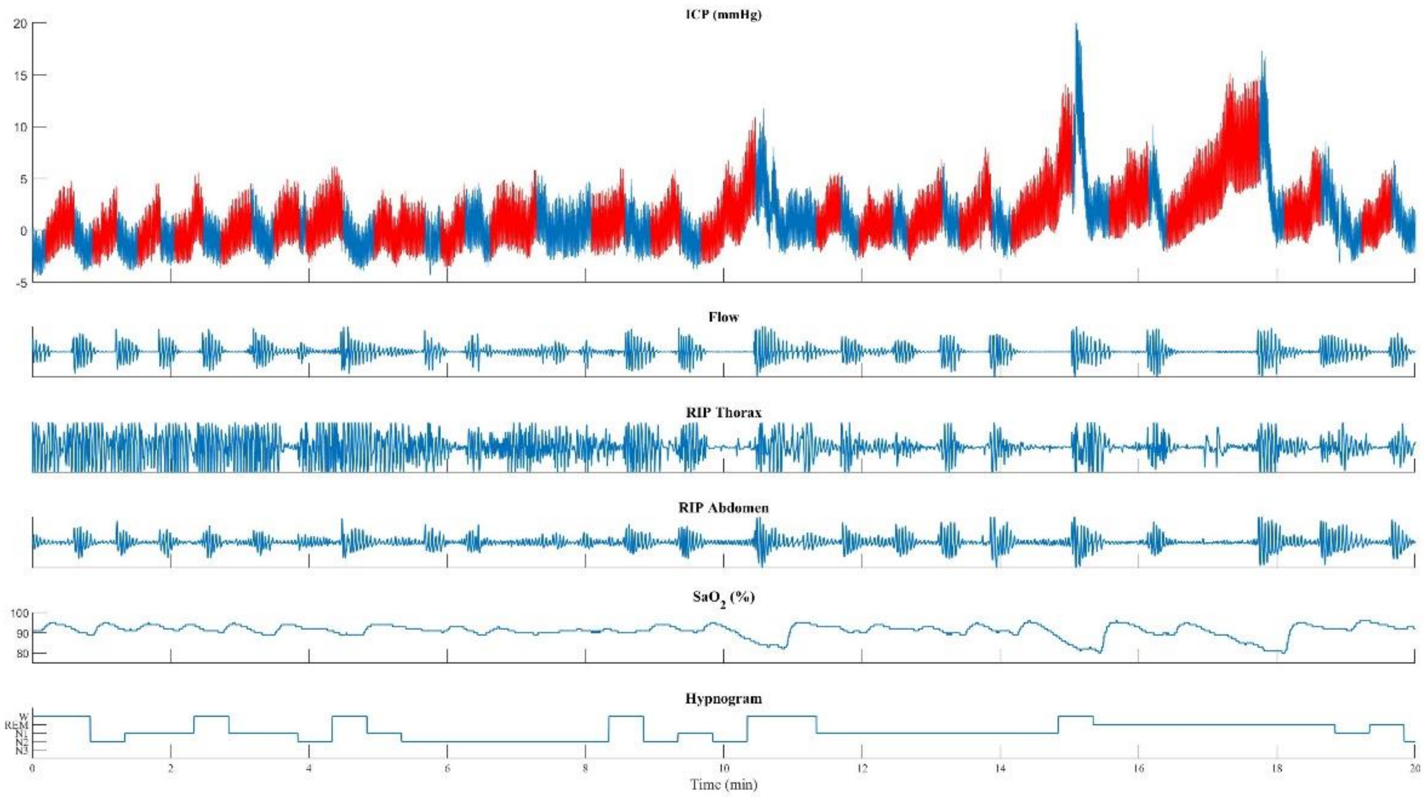

Figure S7 NREM to REM transition in a patient with pediatric-onset hydrocephalus. The peak of the transient ICP elevation is seen when respiration resumes with a ventilatory overshoot, marked by increased flow and respiratory movements in the thorax and abdominal RIP. In NREM, the beginning sinusoidal transient ICP elevation with a low ICP increase and frequent arousals is seen with more activity in the thoracic RIP than in the abdominal RIP. ICP is shown in blue, with red and purple indicating the duration of apneas and respiratory disturbances, respectively. Flow: nasal cannula registering flow changes (arbitrary units). RIP, respiratory inductance plethysmography; thorax and abdomen movements (arbitrary units). SaO_2 (%), oxyhemoglobin saturation measured on the finger. Heart rate, beats/min. Hypnogram with awake and sleep stages.

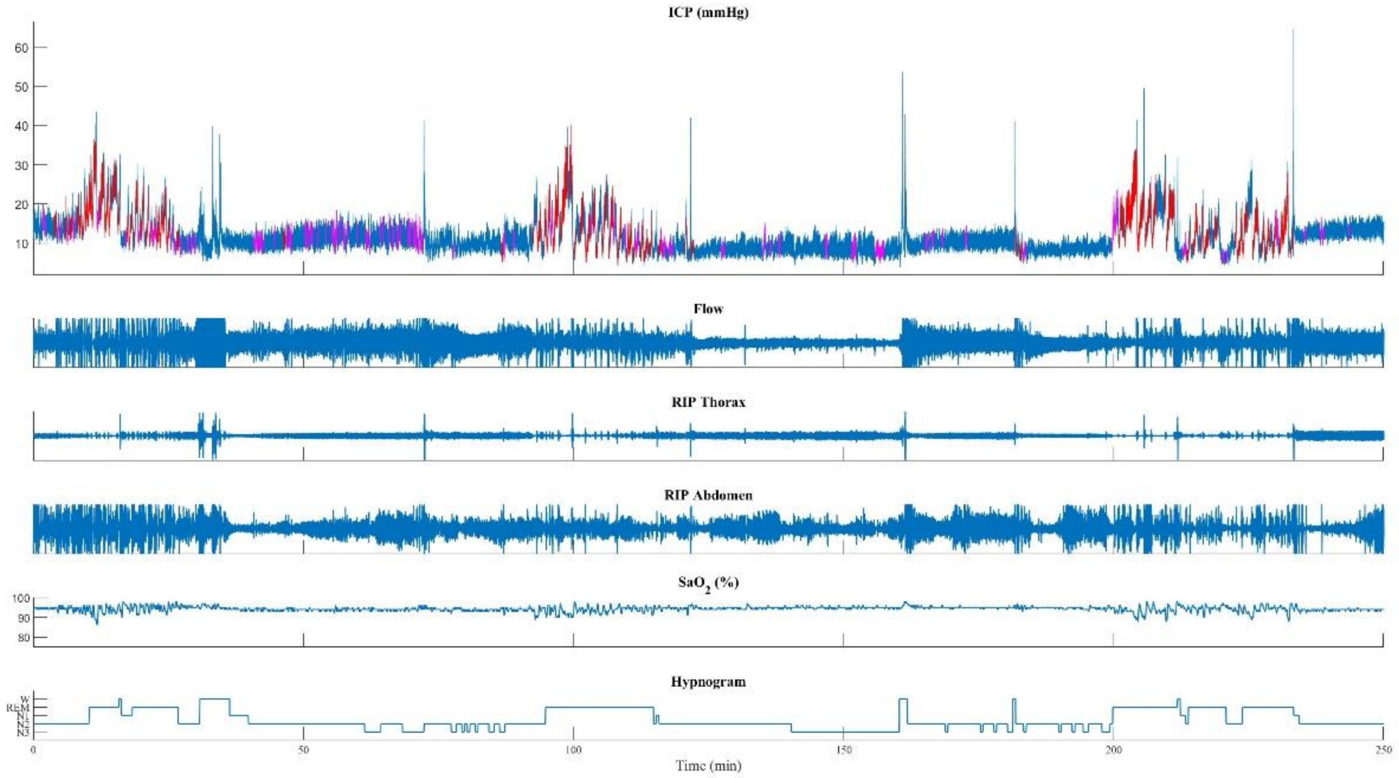

Figure S8 ICP elevations with every REM sleep episode in a patient with IIH. The ICP signal has a declining trend through each REM sleep episode. ICP is blue, with red and purple indicating the duration of apneas and respiratory disturbances. Flow: nasal cannula registering flow changes (arbitrary units). RIP, respiratory inductance plethysmography; thorax and abdomen movements (arbitrary units). SaO_2 (%), oxyhemoglobin saturation measured on the finger. Heart rate, beats/min. Hypnogram with awake and sleep stages.

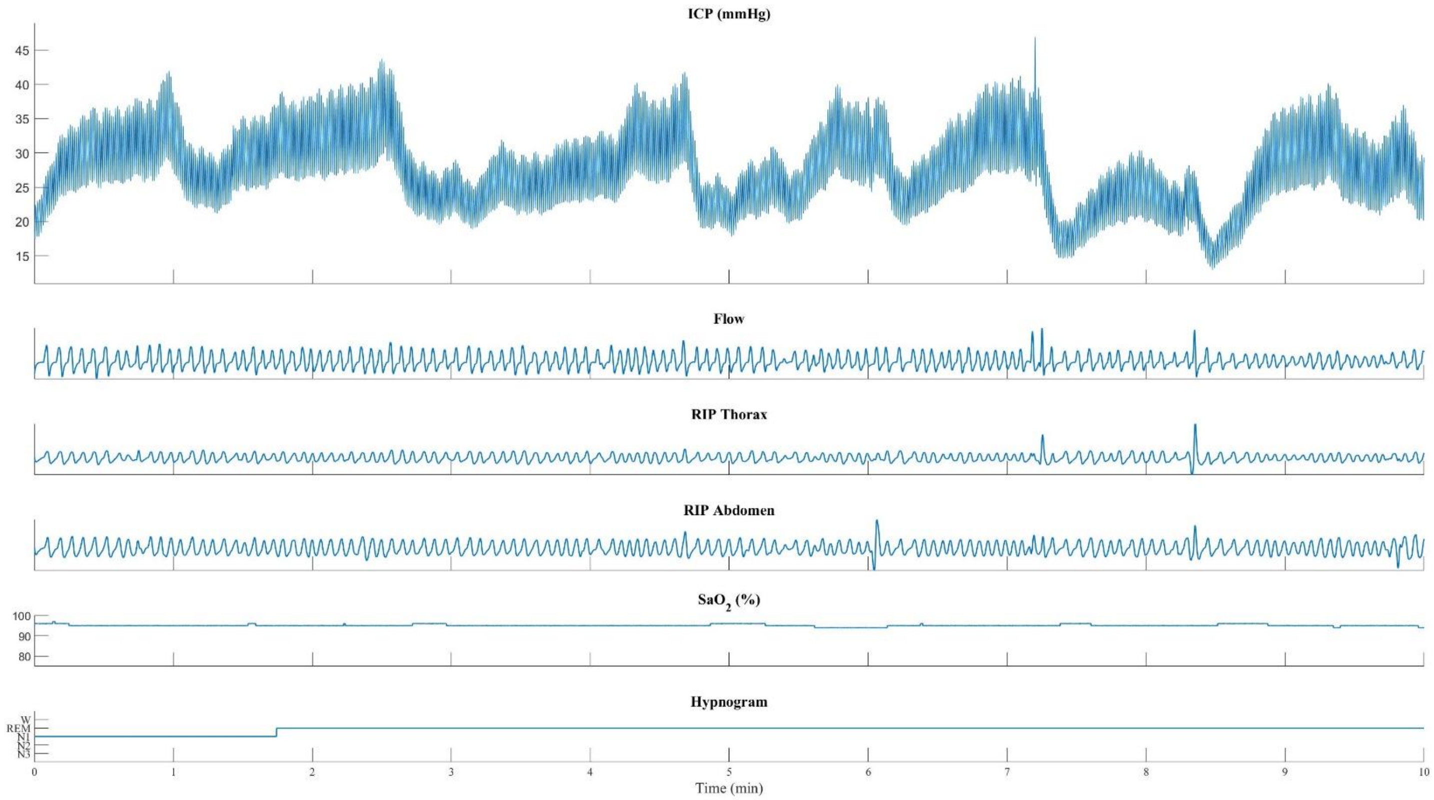

Figure S9 Patient with IIH and repeating ramp-type transient ICP elevations with the onset of REM without apneas, respiratory disturbances, or desaturation. ICP is shown in blue, with red and purple indicating the duration of apneas and respiratory disturbances. Flow, nasal cannula registering flow changes (arbitrary units). RIP, respiratory inductance plethysmography; thorax and abdomen movements (arbitrary units). SaO_2 (%), oxyhemoglobin saturation measured on the finger. Heart rate, beats/min. Hypnogram with awake and sleep stages.

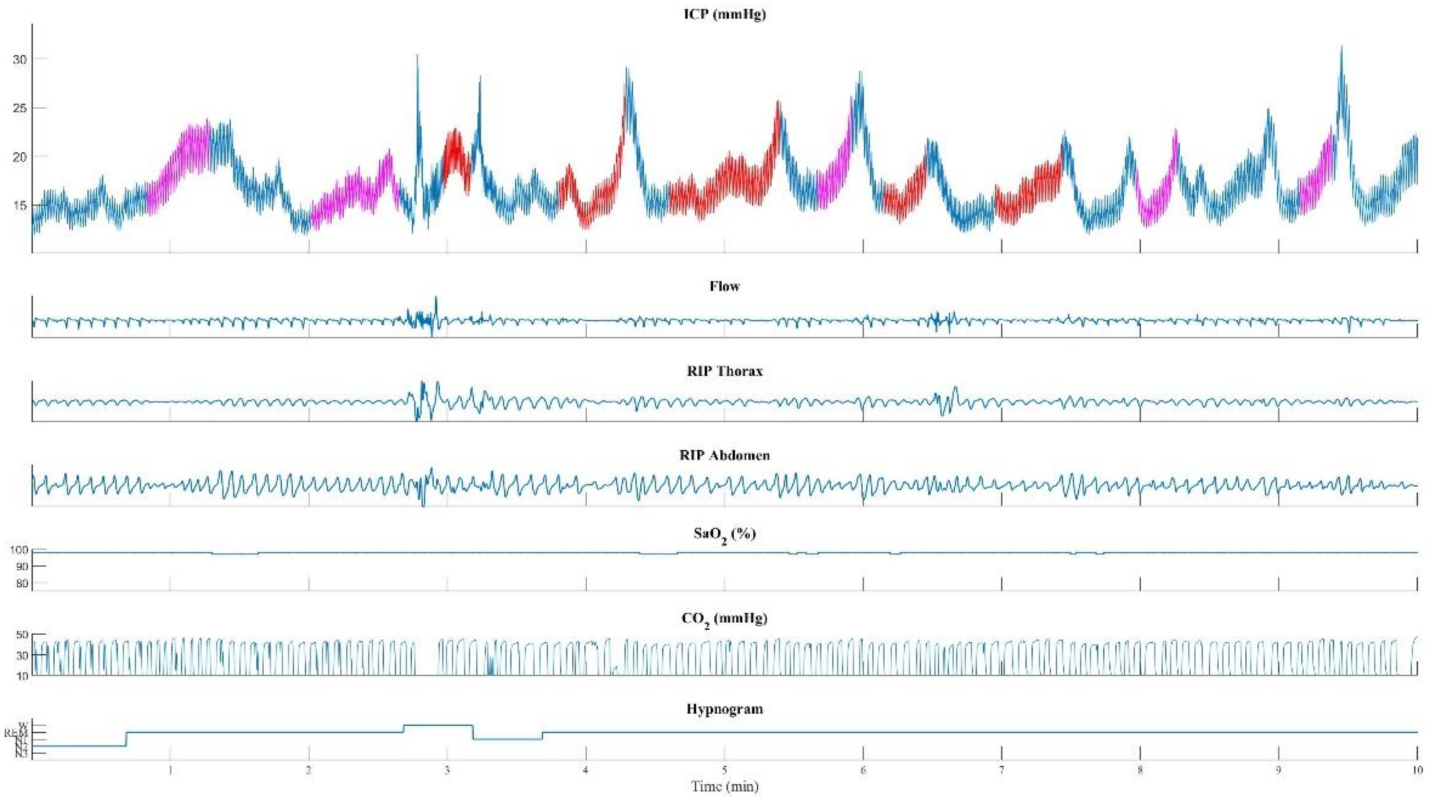

Figure S10 Repeating ramp-type transient ICP elevations with the onset of REM in a patient with IIH without changes in CO_2 and O_2. ICP is blue, with red and purple indicating the duration of apnea and respiratory disturbances. Flow: nasal cannula registering flow changes (arbitrary units). RIP, respiratory inductance plethysmography; thorax and abdomen movements (arbitrary units). SaO_2 (%), oxyhemoglobin saturation measured on the finger. Heart rate, beats/min. Hypnogram with awake and sleep stages. CO_2 was measured with end-tidal LoFLo.

Table S1 Detailed information about all patients

	ICP catheter	Additional information	Number of transient ICP elevations						ICP night		Sleep apnea			
			Apnea	w/o Apnea	NREM	REM	Total	Mean ICP change	Mean (SD)	Duration (min)	AHI	NREM	REM	AH count
iNPH 1	Neurovent		37	312	239	110	349	16.8	7.4 (3.8)	353	26.2	25.2	6.3	75
iNPH 2	Neurovent-P	ETV	71	51	121	1	122	15.6	8.5 (5.1)	494	37.7	37.7	0.0	71
iNPH 3	Neurovent		193	246	439	0	439	13.9	5.8 (3.7)	536	40.2	40.2	0.0	255
iNPH 4	Neurovent		96	283	181	198	379	15.3	8.0 (5.4)	557	40.2	41.3	20.0	124
iNPH 5	Neurovent		229	288	495	22	517	11.6	3.9 (3.4)	1038	30.1	30.9	15.0	357
iNPH 3	Neurovent	CPAP	38	172	210	0	210	19.0	11.4 (5.5)	650	11.2	11.1	0.0	55
iNPH 4	Neurovent	CPAP	105	118	195	28	223	14.0	9.2 (3.8)	327	24.7	28.6	2.9	112
Adult 1	Neurovent	ETV	10	197	179	28	207	16.5	4.0 (3.9)	672	3.2	3.0	6.0	27
Adult 2	Neurovent-P	ETV	21	62	83	0	83	12.9	7.8 (2.3)	557	11.6	11.6	0.0	65
Adult 3	Neurovent-P	ETV	8	283	281	12	291	13.4	7.7 (3.3)	425	1.9	2.0	0.0	9
Adult 4	Neurovent	ETV	23	116	114	25	139	13.9	9.3 (6.5)	455	6.0	4.3	18.8	43
Pediatric 1	Neurovent-P		56	152	177	31	208	12.0	4.9 (3.0)	295	14.2	15.7	1.9	70
Pediatric 2	Neurovent-P		3	381	327	57	384	9.4	2.4 (2.6)	488	1.0	1.1	0.0	8
Pediatric 3	Neurovent-P	ETV	29	620	343	306	649	10.8	5.7 (4.1)	339	8.0	7.9	8.3	45
Pediatric 4	Neurovent-P	ETV	23	515	524	14	538	10.8	4.4 (4.2)	659	4.0	3.4	16.4	40
Pediatric 5	Neurovent-P	ETV	15	308	317	6	323	11.5	14.5 (5.1)	828	0.5	0.5	0.0	7
Pediatric 6	Neurovent-P	ETV	114	310	396	28	424	12.4	13.3 (3.9)	391	21.0	21.2	18.6	134
Pediatric 7	Neurovent	ETV	125	97	212	10	222	11.7	1.9 (3.1)	489	57.6	58.3	34.3	207
Pediatric 8	Neurovent-P	Shunt	0	228	199	29	228	11.2	21.2 (3.9)	556	16.4	13.5	63.5	120
Pediatric 9	Neurovent-P-tel	Shunt + CO_2	9	355	193	170	363	13.4	14.3 (4.7)	634	2.0	2.3	1.2	15,0
Pediatric 10	Neurovent-P	ETV + CO_2	128	259	160	227	387	22.6	16.0 (7.9)	297	29.3	25.5	43.3	123
Pediatric 11	Neurovent-P	Shunt	3	750	725	28	753	16.3	20.2 (5.7)	477	0.5	0.3	2.9	3
Pediatric 12	Neurovent-P	ETV	106	135	163	78	241	14.7	5.3 (3.8)	355	24.8	16.5	50.1	112
Pediatric 13	Neurovent-P		0	452	341	111	452	14.1	10.8 (3.9)	587	0.0	0.0	0.0	0
Pediatric 12	Neurovent-P	CPAP	1	199	83	117	200	15.0	4.3 (4.0)	470	0.2	0.0	0.5	1
IIH 1	Neurovent	Shunt	88	170	205	53	258	19.3	11.0 (10.5)	485	17.9	16.4	28.6	124
IIH 2	Neurovent	Shunt	248	513	679	82	761	21.1	7.3 (9.0)	628	24.6	22.3	39.5	234
IIH 3	Neurovent-P-tel		78	397	326	149	475	12.5	12.5 (4.0)	460	14.1	5.9	44.4	89
IIH 4	Neurovent-P-tel		106	179	248	37	285	21.6	18.7 (5.3)	437	26.2	24.3	48.4	172
IIH 5	Neurovent-P-tel	Shunt	13	295	205	103	308	9.5	8.2 (4.0)	590	3.0	3.4	0.7	27
IIH 6	Neurovent-P	Shunt	37	715	709	43	752	12.1	10.7 (7.1)	631	1.8	1.0	8.6	8
IIH 7	Neurovent-P-tel		26	364	291	110	390	11.4	17.2 (4,3)	569	6.5	6.2	9.1	44
IIH 8	Neurovent-P-tel		37	312	239	110	349	16.8	13.2 (4.3)	442	7.5	4.2	23.6	51
IIH 9	Neurovent-P-tel	CO_2	6	390	255	141	396	22.5	24.3 (8.4)	446	1.3	1.4	0.9	8
IIH 10	Neurovent-P		13	410	398	25	423	31.8	28.6 (11.2)	595	4.0	1.9	19.8	35
IIH 11	Neurovent-P	CO_2	34	334	296	72	368	12.6	14.2 (3.2)	447	10.7	10.4	12.1	57
IIH 12	Neurovent-P	CO_2	3	644	520	127	647	14.1	11.7 (4.7)	598	0.6	0.3	1.6	7

AH = Apnea-Hypopnea; AHI = Apnea-Hypopnea Index; Adult = Adult-onset hydrocephalus; ETV = Endoscopic third ventriculostomy; iNPH = Idiopathic normal pressure hydrocephalus; IIH = Idiophatic intracranial hypertension; Neurovent = Raumedic Neurovent ventricular ICP catether; Neurovent-P = Raumedic Neurovent parenkmal ICP catether; Neurovent-P-tel = Raumedic Neurovent parenkmal telemetric ICP catether; Pediatric = pediatric-onset hydrocephalus; SD = Standard deviation; Shunt = ventriculo-periotoneal shunt

Table S2 Quantification of transient ICP elevations.

	Transient ICP-elevations			Sleep apnea
	Total	Apnea	Without Apnea	AHI
iNPH (*n* = 5)	361 (148)	125 (67)	236 (118)	34.9 (6.4)
Pediatric (*n* = 13)	398 (175)	47 (52)	351 (199)	13.8 (16.8)
IIH (*n* = 12)	451 (171)	57 (70)	394 (151)	9.9 (8.9)
Adult (*n* = 4)	180 (90)	16 (8)	165 (97)	5.7 (4.3)

Transient ICP elevation values are mean and standard deviation (SD). Adult = adult-onset obstructive hydrocephalus; AHI = apnea-hypopnea index; IIH = idiopathic intracranial hypertension; iNPH = Idiopathic normal pressure hydrocephalus; Pediatric = pediatric-onset hydrocephalus; Ratio = the ratio between transient ICP elevation with apnea and without apnea.

Table S3 Continuous positive airway pressure (CPAP) usage in the three patients.

	**CPAP used (hours)**	**Mean pressure CPAP (mmHg)**	**Mean ICP first night (mmHg)**	**Mean ICP with CPAP (mmHg)**
iNPH 1	8.0	6.4	5.8	11.4
iNPH 2	6.0	7.7	8.0	9.2
Pediatric (with VP-shunt)	8.0	7.5	5.3	4.3
Mean	7.3	7.2	6.4	8.3

Mean pressure CPAP indicates the mean pressure applied by the CPAP during the hours it was used. iNPH = Idiopathic normal pressure hydrocephalus; Pediatric = pediatric-onset hydrocephalus; VP-shunt = ventriculoperitoneal shunt
